# A Rarefaction Approach to Identify Local Introgression in a Three Population Tree

**DOI:** 10.64898/2026.05.13.724952

**Published:** 2026-05-15

**Authors:** T. Quinn Smith, Zachary A. Szpiech

**Affiliations:** 1 Department of Biology, The Pennsylvania State University, University Park, PA 16802

## Abstract

Patterson’s *D* statistic, also known as the *ABBA*−*BABA* statistic, is widely used to detect the presence of archaic genome-wide introgression between two non-sister taxa. Requiring only a single lineage from each of four taxa where one taxon acts as an outgroup to determine the ancestral allele, Patterson’s *D*, counts the imbalance between the number of biallelic sites where either the second and third taxa (ABAB site) or the first and third taxa (BABA site). When there is no introgression, these counts are expected to be equal, and a discordance between counts suggests introgression from the third taxon into either the first or second. Patterson’s D is limited to the detection of genome-wide introgression and exhibits a high false-positive rate when applied to smaller genomic segments. Here, we present a new method, D STatistic with Allelic Rarefaction $\left(D^{*}\right)$, to address these limitations. $D^{*}$ uses multiple lineages and does not require an outgroup to calculate the imbalance between the number of alleles found exclusively in the second and third taxa and the number of alleles found exclusively in the first and third taxa. $D^{*}$ employs a rarefaction technique to correct for unequal sample-size and allows multiallelic sites. We use simulations to show that $D^{*}$ has better precision and recall for detecting introgressed segments of DNA when compared to similar methods under a wide variety of model parameters and in the presence of technical artifacts common to ancient DNA analyses. We conclude with an analysis of Denisovan DNA introgression in modern day Papuans. Precompiled executables, the manual, and source code can be found at https://github.com/TQ-Smith/DSTAR

## 1 Introduction

Introgression is the transfer of genetic material between divergent lineages and is a pervasive demographic force that can shape the evolutionary trajectory of species. By introducing novel genetic variation, introgression can alter the genetic composition of populations, facilitate adaptation, and drive the speciation process [1, 2]. Recent genomic studies have highlighted the impact of introgression across human and non-human species, which has left detectable signatures in both modern and ancient populations [3, 4, 5, 6, 7, 8, 9].

Consequently, much effort has been dedicated to identifying and quantifying introgression. While classical population genetics models often struggle to capture the complexities of empirical data [10], and likelihood methods are frequently too computationally intensive to generalize across large sample sizes or multiple populations [11, 12, 13], summary statistics based on allele patterns across taxa are straightforward to compute and highly effective [14]. A classic example of such a statistic is Patterson’s *D*, which can identify the presence of genome-wide introgression within a four-population tree by measuring imbalances in derived allele sharing [15].

However, while Patterson’s *D* is robust when applied to genome-wide data in aggregate, the field’s focus has increasingly shifted toward locating specific, locally introgressed genomic segments [16, 17, 18]. In this context, Patterson’s *D* loses power, particularly in regions of low nucleotide diversity [19]. To address this, window-based adaptations such as $f_{d}$ [19] and *D*^+^ [16], were developed.

Several other methods exist to locate introgressed segments in modern populations; however, such methods exploit linkage disequilibrium (LD) that is eroded over many generations, making them unsuitable to detect archaic signatures [20, 21]. The statistics *S*^∗^ [22] and *SPrime* [8] overcome the reliance on strong LD patterns but are model based and do not scale to many archaic samples. Similarly, likelihood-based methods [23, 24, 2] and approaches using the ancestral-recombination graph (ARG) [25, 26, 27] offer deep insights but sacrifice the computational simplicity of Patterson’s *D*, often scaling poorly with sample size.

The difficulty of identifying local introgressed segments has recently motivated the application of machine learning (ML) classifiers [28, 29, 30, 31]. However, a major limitation of ML is its reliance on extensive simulated training data, which requires *a priori* knowledge of the demographic history of the populations in question [32]. Notably, these ML models consistently identify the number of private alleles within a genomic window as one of the most powerful predictive features for introgression [28, 29, 31].

Private alleles are alleles found exclusively in one population and nowhere else. In the absence of gene flow, divergent populations accumulate private alleles independently; whereas, introgression increases allele sharing and reduces the number of private alleles. Slatkin first formalized the relationship between private alleles and migration rates [33, 34, 35], though he noted that private allele counts are highly sensitive to sample size [34]. Later, Kalinowski [36] applied the rarefaction technique [37] to correct for sample size bias, and this approach was adapted to derive a generalization of private alleles to combinations of populations [38].

Building on the theoretical foundations of Szpiech et al. (2008) [38], we introduce the D STatistic with Allelic Rarefaction $\left(D^{*}\right)$ which combines the reasoning behind $D/D^{+}$ with generalized private alleles and rarefaction to identify the presence of introgression within local regions. While Patterson’s *D* relies on imbalances in derived allele counts at biallelic sites within a four-population tree [15], An imbalance between counts indicates gene flow between populations within a pair. $D^{*}$ generalizes this reasoning to sites with any number of alleles and removes the requirement of an outgroup. Indeed, recent work has demonstrated that outgroups are not strictly necessary for detecting genome-wide introgression [39]. Furthermore, by incorporating allelic rarefaction, $D^{*}$ corrects for unequal sample sizes that might otherwise confound empirical studies.

In this study, we evaluate $D^{*}$ through extensive simulations and compare its performance to *D* and *D*^+^ in locating locally introgressed regions. We assess the statistic’s robustness across a range of evolutionary scenarios, varying the proportion of introgression, mutation rates, and recombination rates, while exploring its behavior under small and unequal sample sizes. Because recent studies have shown that deviations from a strict molecular clock and lineage-specific differences in drift can drastically skew the conclusions drawn from Patterson’s *D* [40], we explicitly investigate the impact of such deviations on $D^{*}$. To ensure its utility for ancient DNA (aDNA) research, we test the performance of $D^{*}$ against common empirical artifacts such as missing genotypes, deamination, and pseudohaploidization. Finally, we apply $D^{*}$ to empirical data to identify previously known and novel Denisovan introgressed tracts in modern-day Papuans.

## 2 Materials and Methods

### 2.1 Definition of Patterson’s D and $D^{+}$

Consider a set of four taxa, $\left\{P_{1},P_{2},P_{3},P_{4}\right\}$ with a divergence relationship of $\left(\left(\left(P_{1},P_{2}\right),P_{3}\right),P_{4}\right)$, indicating that $P_{1}$ and $P_{2}$ split after divergence from $P_{3}$ and $P_{4}$ is an outgroup. Considering only biallelic sites, let *A* denote the ancestral allele and let *B* denote the derived allele. At the $l^{th}$ locus, we define the indicator variables $C_{ABBA}(l)$, $C_{BABA}(l)$, $C_{BAAA}(l)$, and $C_{ABAA}(l)$, to indicate the allele patterns (((A,B),B),A),(((B,A),B),A),(((B,A),A),A), and (((A,B),A),A) across the four taxa, respectively. Over *L* sites, Patterson’s *D* is defined as

(1) $$D=\frac{\sum_{l=1}^{L}C_{ABBA}(l)-C_{BABA}(l)}{\sum_{l=1}^{L}C_{ABBA}(l)+C_{BABA}(l)}$$

In the absence of introgression, *D* has an expected value of 0. If introgression occurred between $P_{2}$ and $P_{3}$, *D* is expected to be greater than 0. If introgression occurred between $P_{1}$ and $P_{3}$, *D* is expected to be less than 0 [15]. As the power of *D* to detect introgression in short segments, especially those with low nucleotide diversity, is weak [19], *D*^+^ [16] was developed to overcome this shortcoming. *D*^+^ is defined as

(2) $$D^{+}=\frac{\sum_{l=1}^{L}\left(C_{ABBA}(l)-C_{BABA}(l)\right)+\left(C_{BAAA}(l)-C_{ABAA}(l)\right)}{\sum_{l=1}^{L}\left(C_{ABBA}(l)+C_{BABA}(l)\right)+\left(C_{BAAA}(l)+C_{ABAA}(l)\right)}$$

The interpretation of *D*^+^ is identical to *D*. If multiple lineages are available, let *p*_*li*_ be the derived allele frequency at the $l^{th}$ site in the $i^{th}$ population. Then, *D* and *D*^+^ are defined as

(3) $$D=\frac{\sum_{l=1}^{L}\left(1-p_{l1}\right)p_{l2}p_{l3}\left(1-p_{l4}\right)-p_{l1}\left(1-p_{l2}\right)p_{l3}\left(1-p_{l4}\right)}{\sum_{l=1}^{L}\left(1-p_{l1}\right)p_{l2}p_{l3}\left(1-p_{l4}\right)+p_{l1}\left(1-p_{l2}\right)p_{l3}\left(1-p_{l4}\right)}$$

and

(4) $$D^{+}=\frac{\sum_{l=1}^{L}\left(\left(1-p_{l1}\right)p_{l2}p_{l3}\left(1-p_{l4}\right)-p_{l1}\left(1-p_{l2}\right)p_{l3}\left(1-p_{l4}\right)\right)+\left(p_{l1}\left(1-p_{l2}\right)\left(1-p_{l3}\right)\left(1-p_{l4}\right)-\left(1-p_{l1}\right)p_{l2}\left(1-p_{l3}\right)\left(1-p_{l4}\right)\right)}{\sum_{l=1}^{L}\left(\left(1-p_{l1}\right)p_{l2}p_{l3}\left(1-p_{l4}\right)+p_{l1}\left(1-p_{l2}\right)p_{l3}\left(1-p_{l4}\right)\right)+\left(p_{l1}\left(1-p_{l2}\right)\left(1-p_{l3}\right)\left(1-p_{l4}\right)+\left(1-p_{l1}\right)p_{l2}\left(1-p_{l3}\right)\left(1-p_{l4}\right)\right)}$$

### 2.2 Counting Alleles

We follow the approach of Szpiech et al. [38] for counting alleles ”private to combinations of populations”, that is, for counting alleles found in each of a set of populations and in no other population outside of that set. At a locus with I distinct alleles, we sample $N_{ij}$ copies of allele i from population j, where $N_{j}=\sum_{i=1}^{I}N_{ij}$ is the total number of lineages in population j.

We define the probability of *not* observing a copy of allele *i* from a subsample of size g lineages from population j as

(5) $$X_{ij}^{g}=\frac{\left(\begin{array}{c} N_{j}-N_{ij}\\ g \end{array}\right)}{\left(\begin{array}{c} N_{j}\\ g \end{array}\right)}$$

We calculate the probability of observing *at least* one copy of allele i from a subsample of size g lineages from population j as $Y_{ij}^{g}=1-X_{ij}^{g}$. Then the number of alleles found in both $P_{1}$ and $P_{3}$ but absent in $P_{2}$ in a subsample of size g lineages is given by

(6) $$\pi_{P_{1},P_{3}}^{g}=\sum_{i=1}^{I}Y_{i1}^{g}X_{i2}^{g}Y_{i3}^{g}.$$

We call this term the number of alleles private to $P_{1}$ and $P_{3}$.

Similarly, the number of alleles common to $P_{2}$ and $P_{3}$ but absent in $P_{1}$ in a subsample of size g lineages is given by

(7) $$\pi_{P_{2},P_{3}}^{g}=\sum_{i=1}^{I}X_{i1}^{g}Y_{i2}^{g}Y_{i3}^{g}.$$

We call this term the number of alleles private to $P_{2}$ and $P_{3}$.

We also compute the total number of alleles at a locus (allelic richness) for populations $P_{1}$, $P_{2}$ and $P_{3}$ combined. Let $N=N_{1}+N_{2}+N_{3}$, then, for each allele, the probability of observing *at least* one copy of allele i in a sample size of g is $1-\frac{\left(\begin{array}{c} N-N_{i1}-N_{i2}-N_{i3}\\ g \end{array}\right)}{\left(\begin{array}{c} N\\ g \end{array}\right)}$. The total number of alleles at a locus is then given by

(8) $$\kappa^{g}=\sum_{i=1}^{I}\left[1-\frac{\left(\begin{array}{c} N-N_{i1}-N_{i2}-N_{i3}\\ g \end{array}\right)}{\left(\begin{array}{c} N\\ g \end{array}\right)}\right].$$

### 2.3 Definition of $D^{*}$

Let $\pi_{P_{2},P_{3}}^{g}(l)$, $\pi_{P_{1},P_{3}}^{g}(l)$, and $\kappa^{g}(l)$ be the statistics defined in Section 2.1 calculated at a locus l. We define $D^{*}$ over L loci as

(9) $$D^{*}=\frac{\sum_{l=1}^{L}\left(\pi_{P_{2},P_{3}}^{g}(l)-\pi_{P_{1},P_{3}}^{g}(l)\right)}{\sum_{l=1}^{L}\kappa^{g}(l)},$$

which contrasts the number of alleles private to $P_{1}$ and $P_{3}$ to the number of alleles private to $P_{2}$ and $P_{3}$, normalized by the allelic richness of the three populations combined.

In the case of no introgression from $P_{3}$ *, $P_{1}$ and $P_{2}$* should share approximately equal amounts of alleles private with $P_{3}$, that is those inherited from the common ancestor of all three populations but lost in either $P_{1}$ *or $P_{2}$*. In this case we expect $\pi_{P_{2},P_{3}}^{g}=\pi_{P_{1},P_{3}}^{g}$ and $D^{*}=0$. In the case of gene flow between $P_{3}$ and $P_{1}$, we expect more alleles private to $P_{3}$ and $P_{1}$ relative to the number of alleles private to $P_{3}$ and $P_{2}$ and thus expect $\pi_{P_{1},P_{3}}^{g}>\pi_{P_{2},P_{3}}^{g}$ and $D^{*}<0$. In the case of gene flow between $P_{3}$ and $P_{2}$, we expect more alleles private to $P_{3}$ and $P_{2}$ relative to the number private to $P_{3}$ and $P_{1}$, thus $\pi_{P_{2},P_{3}}^{g}>\pi_{P_{1},P_{3}}^{g}$, and $D^{*}>0$.

We only calculate Equation 9 for sites where $\pi_{P_{2},P_{3}}(l)>0$ or $\pi_{P_{1},P_{3}}(l)>0$, as including sites that are private to neither pair artificially deflates $D^{*}$. The parameter, g, is set to the minimum number of non-missing lineages within one of the three populations at a given site. $D^{*}$ is calculated in non-overlapping genomic windows measured in base pairs.

### 2.4 Simulations

**Figure 1:**
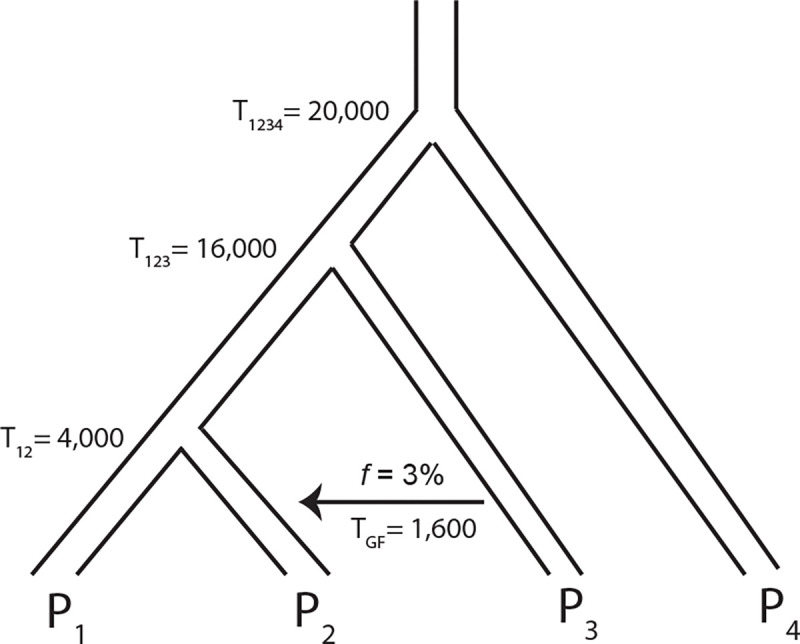
Simulated Demographic Scenario. The simplified demographic history used to evaluate $D^{*}$. Divergence times are in generations. By default, all populations have $N_{e}=10,000$.

We used *msprime* [41] to simulate a simplified demographic history (Figure 1) as a benchmark to model archaic introgression in modern humans, following previous work [42, 16]. We treated $P_{1}$ and $P_{2}$ as modern populations and $P_{3}$ as the population that exchanges lineages with $P_{2}$. We set the admixture proportion between $P_{3}$ and $P_{2}$ to f=3% occurring at $T_{GF}=1,600$ generations ago. The divergence times between $P_{1}$ and $P_{2}$ is $T_{12}=4,000$ generations ago. The ancestral population, $P_{3}$ diverged from $P_{1}$ and $P_{2}$ $T_{123}=16,000$ generations ago. We added an outgroup, $P_{4}$, which diverged from the other three populations $T_{1234}=20,000$ generations ago. The effective population size for all populations was set to $N_{e}=10,000$. We set the length of the genomic region to 20 Mbp, the mutation rate to $\mu =1.5\times 10^{-8}$ per base pair per generation, and the recombination rate to $\rho =1\times 10^{-8}$. We computed D, $D^{+}$, and $D^{*}$ with this demographic history using a single lineage sampled from the four populations.

To explore the influence of admixture proportion, mutation rate, and recombination rate we vary each within the following ranges, f={3%,5%,10%}, $\mu =\left\{0.75\times 10^{-8},1.5\times 10^{-8},3.0\times 10^{-8}\right\}$, and $rho=\left\{0.5\times 10^{-8},1\times 10^{-8},1.5\times 10^{-8}\right\}$, creating 27 parameter combinations.

To test the effects of uneven sample sizes, we set f=3%, $\mu =1.5\times 10^{-8}$, and $\rho =1\times 10^{-8}$. Then we sampled 25 lineages from $P_{1}$, 2 lineages from $P_{3}$, and 2 lineages from $P_{4}$, and sampled $N_{2}=\{2,5,10,25\}$ lineages from $P_{2}$. For all four sample sizes, g=2 since each population contains at least 2 lineages.

For all parameter combinations, 100 replicates with and without introgression (f=0) were generated. The replicates without introgression are treated as a null model for performance evaluation and further described in Section 2.7.

### 2.5 Molecular Clock Violations and Effects of Drift

An assumption of our simplified demographic model is the use of a constant mutation rate across all populations. Often, mutation rates vary between species, and therefore, affect the accumulation of derived alleles within a population [43]. Deviations in the molecular clock have been shown to influence conclusions using Patterson’s *D* [40]. *msprime* is not capable of varying mutation rates between populations directly. Instead, we mimic the uneven accumulation of mutations within a population by rescaling divergence times and sampling times [16]. Generally, if we want to scale the mutation rate of $P_{2}$ by a factor λ, we increase all divergence times and $T_{GF}$ by $(\lambda -1)T_{12}$. Then, we sample $P_{2}$ at time 0 and all other populations $(\lambda -1)T_{12}$ generations ago. We can swap the roles of $P_{1}$ and $P_{2}$ to equivalently increase the mutation rate of $P_{1}$ by a factor of λ. These scenarios were evaluated with a single lineage sampled from each population and considered λ={1.2,1.5,2,5} for $P_{1}$ and $P_{2}$. [40].

To test the effect of different levels of drift between populations, in particular in the effects of small effective population, we varied $N_{e}=\{2500,5000,25000\}$ for $P_{1}$ and $P_{2}$, creating six different scenarios. We kept the other populations’ effective population size at $N_{e}=10000$. These scenarios were also evaluated with a single lineage sampled from each population.

### 2.6 Missing Data, Deamination, and Pseudohaploidization

Simulated data do not contain sources of error known to bias inference [44, 45]. We also simulate several sources of error on top of our simulated genomic region that are common when working with modern and ancient DNA. We used parameters gathered from ancient DNA studies to maintain a sense of realism and act as a baseline for other studies.

The low quality of aDNA prevents many alleles from being accurately called, which leads to a large amount of missing data. When an allele can be called, the coverage is often too low to confidently call a second allele [46]. For aDNA samples that are possibly heterozygous at a site, a random allele is chosen from the reads, and the sample is forced to be homozygous for the random allele. This process is known as pseudohaploidization [47]. In addition, aDNA is particularly prone to the deamination of cysteine to thymine [45, 48]. We investigated the influence of all three processes on $D^{*}$.

We used the simplified demographic history and sampled 10 diploid individuals from $P_{1}$, 10 diploid individuals from $P_{2}$, 2 diploid individuals from $P_{3}$, and one diploid individual from $P_{4}$. A sample’s genotype is missing at a site if both alleles are absent (./.). We introduced missing genotypes according to the process described by Pandey et al [49]. Define a *β*-distribution with a mean of 0.55 and a standard deviation of 0.23. For each sample’s genotype, we drew a random number, b, from our *β*-distribution to determine the proportion of individuals that will be treated as missing at the given site. We randomly set both alleles missing (./.) for ⌊23*b*⌋ of the individuals. We introduced deamination according to Harney et al [48] and treated a locus as a transition with a probability of 77.6%. For each alternative allele, we switched it to the reference allele with a probability of 5%. Then, pseudohaploids were created in the dataset as follows. For each heterozygous individual, we randomly selected one of the alleles with equal probability to make the individual homozygous for that allele [47]. We investigated the effects of all three mechanisms separately and together. Missing genotypes, deamination, and pseudohaploization were introduced into the replicates using the EGGS software [50].

### 2.7 Performance Evaluation

We evaluated D, $D^{+}$, and $D^{*}$ in 50 Kb, non-overlapping blocks for all replicates. The block size of 50 Kb was chosen to reflect the average length of an archaically introgressed haplotype in our simplified demographic model [30]. If a statistic was undefined for any block, then the block was discarded. For each simulation scenario, we generated 100 replicates without gene flow (f=0) to create null-distributions for the three statistics. The null-distributions were used to calibrate false-positive rates (FPRs). We treated 0<α<1 as our significance level. Since we are testing introgression between $P_{2}$ and $P_{3}$, we define our significance threshold at the top $100\frac{\alpha }{2}\text{\%}$ of values for the given statistic.

A true positive (TP) is a block that is statistically significant and introgressed from $P_{3}$. In the case of sampling single lineages, we label a block as introgressed in the event that the lineage from $P_{2}$ contains a total of 5 Kb from $P_{3}$. In the case of sampling multiple lineages, we label a block as introgressed by satisfying two conditions. The first condition is that 10% of the sampled lineages must share a common segment from $P_{3}$. The second condition is that the sum total of these shared segments must span at least 10% of the block (5 Kb). These requirements are identical to Fang et al [16]. A false negative (FN) is a block that was introgressed but not deemed statistically significant. A false positive (FP) is a block that was not introgressed but deemed statistically significant. We measure precision as $\frac{TP}{TP+FP}$, which is the probability that a block was introgressed and significant out of all significant blocks. We measure recall as $\frac{TP}{TP+FN}$, which is the probability that a block was introgressed and significant out of all introgressed blocks.

### 2.8 Genome-Wide Introgression

Although the intent for $D^{*}$ is to identify possibly introgressed local segments, it is worth investigating $D^{*}$ ’s ability to indicate genome-wide introgression. Our approach implements a block bootstrap to test the statistical significance of the genome-wide signal, unlike previous methods based on the rarefaction of private alleles [36, 38]. Another statistic similar to Patterson’s D, $D_{3}$, can infer the presence of genome-wide introgression without an outgroup, but it does not allow multiple lineages sampled from $P_{1}$, $P_{2}$, and $P_{3}$ [39]. $D^{*}$ can accommodate multiple lineages sampled from the three populations in addition to incorporating multiallelic sites.

The significance of D, $D^{+}$, and $D^{*}$ was calculated using a block bootstrap with 1000 replicates [39]. For each statistic, the mean and standard deviation were calculated and used to parameterize a *z*-distribution to calculate significance. We set a significance threshold of *p* < 0.05 to determine the power of each statistic. The ability of $D^{*}$ to signal genome-wide introgression was evaluated for the case of N=1 and N={2,5,10,25} described in Section 2.4.

### 2.9 Denisovan Introgression in Papuans

There is extensive evidence of introgression from Denisovans into modern-day Papuan individuals [51, 52, 23, 53]. We used $D^{*}$ to explore possibly introgressed Denisovan segments in Papuan individuals. Modern-day Sardinians possess low amounts of admixture with Papuans and are more recently diverged from Papuans than Denisovans [52]. This results in the tree ((*Sardinians, Papuans*)*, Denisovans*). Here, Sardinians, Papuans, and Denisovans act as $P_{1}$, $P_{2}$, and $P_{3}$, respectively.

We retrieved the Denisovan sample from Meyer et al [51] that was later reprocessed in Prufer et al [54] from v62 of the Allen Ancient DNA Resource [55]. We converted the dataset to Variant Call Format using EGGS [50]. The sample’s variants were lifted over from hg19 to hg38. Then, we extracted the 27 Sardinian individuals and the 17 Papuan individuals from the whole-genome sequences of the Human Genome Diversity Project (HGDP) [56]. These samples were merged with the Denisovan sample using bcftools [57]. Only biallelic autosomal variants were included in the analysis.

In addition to the set of all samples, it is worth evaluating $D^{*}$ using single lineages from each population. The samples from the HGDP are phased and the Denisovan sample is pseudohaploid. We extracted a random single lineage from the Sardinian population, a random single lineage from the Papuan population, and collapsed the Denisovan sample into a single haploid sample. We evaluated $D^{*}$ and $D^{+}$ on the set of all samples and the set of single lineages in 50 Kb blocks. For each site, we used the reference allele as the outgroup state when calculating $D^{+}$.

## 3 Results

### 3.1 Performance under Simplified Demographic Scenario

For 100 replicates, we calculated D, $D^{+}$, and $D^{*}$ in 50 Kb blocks along simulated 20 Mb segments by sampling a single lineage from each population generated under the demographic scenario in Figure 1 with an admixture proportion of f=0. We used this to create a null-distribution for each of the statistics (Figures SS1a SS1b). The null-distributions for $D^{+}$ and $D^{*}$ are similar in mean and shape, except that the standard deviation of $D^{*}$ (0.1838) is slightly lower than the standard deviation of $D^{+}$ (0.1894). Both are lower than the standard deviation of D (0.6787). This is desirable since a narrower null-distribution will lower the FPR of $D^{+}$ and $D^{*}$ compared to that of *D*. We see this in the false-positive curves of the statistics (Figures SS1c and SS1d) plotted for a range of significance levels (0<α<1). The FPRs for $D^{+}$ and $D^{*}$ are similar over the range of significance levels. At an α=0.05, we found the FPR of $D^{+}$ was 0.0506 and the FPR of $D^{*}$ was 0.0509, which is less than the FPR of D (0.3854). It should be noted that fewer blocks were dropped when calculating $D^{+}$ and $D^{*}$ compared to D. This contributes to $D^{+}$ and $D^{*}$ having a lower FPR. In addition, we focus primarily on the significance levels 0.01≤α≤0.05 as they would correspond to realistic empirical thresholds.

**Figure 2:**
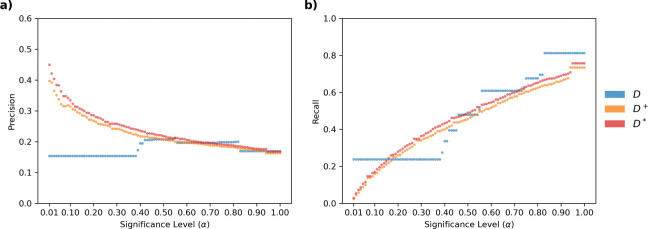
Precision and Recall for Single Sampled Lineages Admixture proportion of f=0.03 was used. All statistics were computed in 50,000 Bp non-overlapping blocks. **Panel a)** Precision and **Panel b)** recall are shown for significance levels (α) between 0.01 and 1.

We next compute the precision and recall for the three statistics with an admixture proportion of f=3% (Figure 2). $D^{*}$ has better precision and recall than $D^{+}$ for α≤0.05. At α=0.05, the precision and recall for $D^{*}$ is 38.29% and 10.72%, respectively, compared to the precision and recall for $D^{+}$ (33.79% and 8.99%). The precision of D is 15.39% in 0.01≤α≤0.05. D shows better recall (23.81%) in this interval than both $D^{*}$ and $D^{+}$.

We calculated the precision and recall calculations with different mutation rates, recombination rates, and admixture proportions. Changing the mutation rate affects the amount of mutations that accumulate in the simulated segments, changing the recombination rate affects the length of introgressed segments, and changing the admixture proportion affects the number of lineages exchanged between $P_{2}$ and $P_{3}$. We investigated their interplay by analyzing each combination of f={3%,5%,10%}, $\mu =\left\{0.75\times 10^{-8},1.5\times 10^{-8},3.0\times 10^{-8}\right\}$, and $\rho =\left\{0.5\times 10^{-8},1\times 10^{-8},1.5\times 10^{-8}\right\}$.

We show the precision and recall for each combination in Figures SS2 SS3, SS4, SS5, SS6, and SS7 and see general trends across the combinations. Both $D^{*}$ and $D^{+}$ perform similarly in terms precision and recall. The precision of $D^{*}$ and $D^{+}$ is better than the precision of *D*, but the recall of *D* is better than the recall of $D^{*}$ and $D^{+}$ with some exceptions. Precision is more sensitive to the parameters than recall. $D^{*}$’s precision range in the parameter space is from 12.70% to 89.23% (Figure SS2a and Figure SS4i) while the recall range is from 2.30% to 16.58% (Figure SS6a and Figure SS7i) at α=0.01. Admixture proportion has the greatest influence on precision compared to mutation rate. At α=0.05, increasing the admixture proportion from f=5% to f=10% results in increasing the precision at most by 21.87% (Figures SS3f and SS4f). A similar doubling of mutation rate, for $\rho =0.5\times 10^{-8}$ and f=10% at α=0.05, increased precision from 52.40% to 63.16% (Figures SS4a and SS4d).

A decrease in recombination rate results in larger introgressed segments, and therefore, an increase in precision. This increase is slight compared to the influence of admixture proportion and mutation rate. When the recombination rate is halved from $\rho =1\times 10^{-8}$ to $\rho =0.5\times 10^{-8}$ at $\mu =3\times 10^{-8}$ and α=0.05, $D^{*}$’s precision increases from 20.83% to 24.00% (Figures SS2b and SS2a). The increase in recombination rate consistently decreases the recall of D (Figures SS5, SS6, and SS7). The shorter introgressed segments cause an increase in false-negatives since these short segments do not contain enough ABBA−BABA sites to correctly classify them as introgressed. While the recall of D is typically better at the significance levels of interest compared to $D^{*}$ and $D^{+}$, the higher recombination rate results in the opposite. At α=0.05, $\mu =3\times 10^{-8}$, and $\rho =2\times 10^{-8}$, $D^{*}$ ’s recall is 39.46%, 55.33%, and 72.80% compared to D ’s recall of 8.05%, 13.60%, and 26.04%, for f=3% 5%, 10%, respectively.

Next, we tested the effects of uneven sample sizes between the three populations. In the simplified demographic model (Figure 1), we sampled 25 lineages from $P_{1}$, N={2,5,10,25} lineages from $P_{2}$, and 2 lineages from $P_{3}$. We show the null distributions for each N in Figure SS8. The statistics’ null distributions become narrower as *N* increases. For N=2, the standard deviation of $D^{*}$ ’s null-distribution is 0.0452, and the standard deviation of $D^{+}$ ’s null-distribution is 0.1124 (Figures SS8a and SS8b). For N=25, the standard deviation of $D^{*}$ ’s null-distribution is 0.0179, and the standard deviation of $D^{+}$ ’s null-distribution is 0.07962 (Figures SS8g and SS8h). The addition of an lineage from N=1 to N=2 has a greater impact on $D^{*}$ than $D^{+}$ (Figures S1a and S1b). This effect diminishes with more sampled lineages, but $D^{*}$ ’s null-distribution remains to be narrower than $D^{+}$ ’s null-distribution.

**Figure 3:**
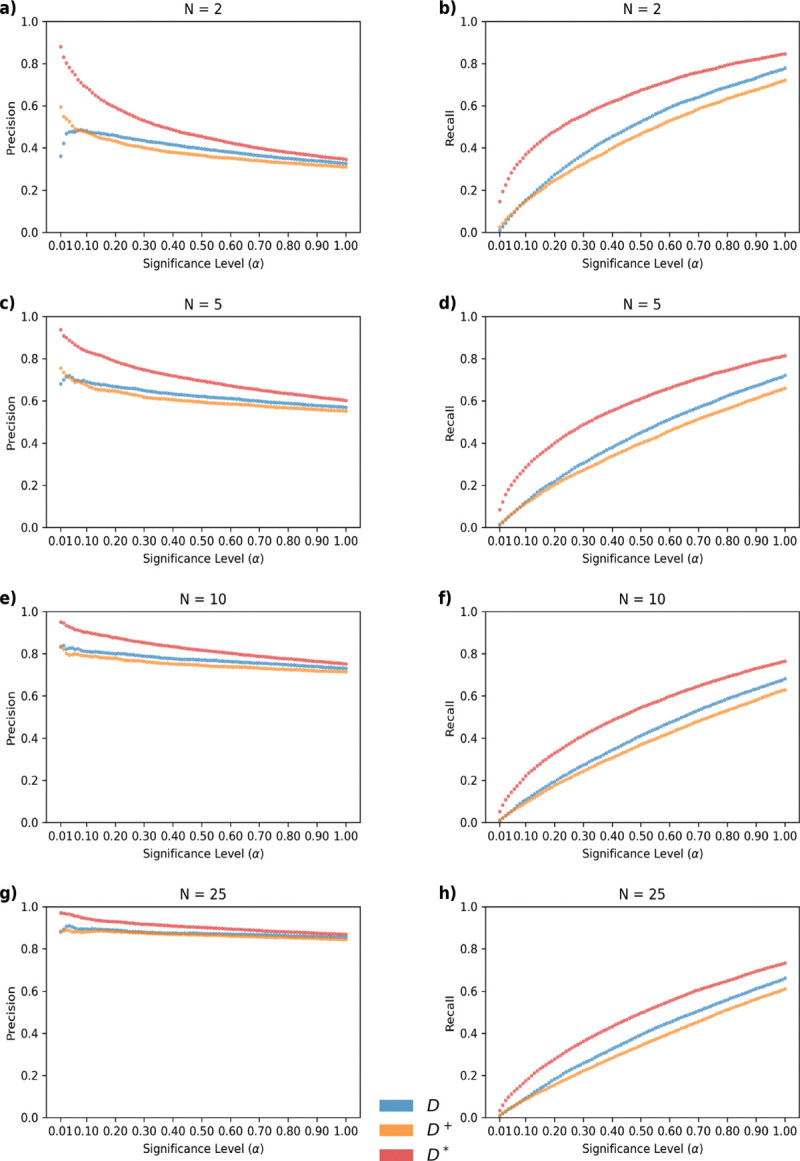
Precision and Recall for Multiple Sampled Lineages Admixture proportion of f=0.03 was used. All statistics were computed in 50,000 Bp non-overlapping blocks. 25 lineages were sampled from $P_{1}$, 2 lineages were samples from $P_{3}$, and 2 lineages were sampled from $P_{4}$. The number of lineages sampled from $P_{2}$ were varied for N={2,5,10,25}. **Panel a)** Precision and **Panel b)** recall for N=2. **Panel c)** Precision and **Panel d)** recall for N=5. **Panel e)** Precision and **Panel f)** recall for N=10. **Panel g)** Precision and **Panel h)** recall for N=25.

The tighter null-distribution of $D^{*}$ will cause a lower false-positive rate, and therefore, higher precision compared to $D^{+}$. This is reflected in Figure 3, which shows the precision and recall curves for the statistics with N={2,5,10,25}. For N=2 at α=0.05 (Figures 3a and 3b), $D^{*}$’s precision is 76.31% while $D^{+}$’s precision is 50.41%. This trend is observed as N increases. For N=25 at α=0.05 (Figures 3g and 3h), the difference in precision is not as great between $D^{*}$ and $D^{+}$, but $D^{*}$’s precision (96.13%) is still greater than the precision of $D^{+}$ (88.11%). $D^{*}$ ’s recall remains to be better than D and $D^{+}$ as N increases. At α=0.05, the difference is greatest for N=2, where the recall for $D^{*}$ *, $D^{+}$*, and D is 28.26%, 8.52%, and 7.92%, respectively. The statistics’ recall diminished as sample size increases. At α=0.05 and N=25, the recall for $D^{*}$ *, $D^{+}$*, and D is 11.12%, 4.97%, and 4.65%, respectively.

### 3.2 Performance under Molecular Clock Violations and Varying Drift

Two assumptions of the model presented in Figure 1 are the same mutation rate, known as the molecular clock, and the same amount of genetic drift within each population. First, we tested the effects of molecular clock violations. We did this by rescaling the mutation rate of $P_{1}$ with respect to the mutation rate of $P_{2}$ by scaling factors of λ={1.2,1.5,2,5} and vice-versa (Figures SS9 and SS10). Increasing the mutation rate of $P_{1}$ by a factor of λ=1.2 slightly decreased the precision, at α=0.05, of $D^{*}$ to 36.67% and $D^{+}$ to 33.28% (Figures SS9a and SS9b) compared to 38.29% and 33.79% for the unscaled case (Figure 2). However, scaling the mutation rate by a factor of λ=5, had a more dramatic effect on precision, at α=0.05. The precision decreased to 28.04% for $D^{*}$ and to 24.22% for $D^{+}$ (Figures SS9g and SS9h).

Scaling the mutation rate of $P_{2}$ by a factor of λ=5, at α=0.05, increased the precision to 39.59% for $D^{*}$ and to 37.76% for $D^{+}$ (Figures SS10g and SS10h) compared to 38.29% and 33.79%, respectively (Figure 2). We see that a small increase in mutation rate with respect to either population has little effect on the precision of $D^{*}$ and $D^{+}$. A larger increase in mutation rate, by a factor of λ=5, for either population, has a more noticeable effect. Scaling up the mutation rate of $P_{1}$ slightly decreased precision for $D^{*}$ and $D^{+}$ while scaling up the mutation rate of $P_{2}$ slightly increased precision. This imbalance could be explained by an increased mutation rate in either $P_{1}$ *or $P_{2}$* raising the probability of either population sharing mutations with $P_{3}$.

We tested the effects of unequal drift by varying the effective population sizes for $P_{1}$ and $P_{2}$ and sampling a single lineage from each of the populations. We tested the effective population sizes $N_{e}=\{2500,5000,25000\}$ for $P_{1}$ (Figure SS9) and $P_{2}$ (Figure SS10). For $P_{1}$ ’s $N_{e}=2500$ at α=0.05, $D^{*}$’s precision was 36.67% and $D^{+}$’s precision was 33.28% (Figures SS9a and SS9b). If we scale $N_{e}$ by a factor of 10 to 25000, then the precision decreased for $D^{*}$ to 33.42% and for $D^{+}$ to 29.22% (Figures SS9e and SS9f). For $P_{2}$ ’s $N_{e}=2500$ at α=0.05, $D^{*}$’s precision was 36.32% and $D^{+}$’s precision was 33.54% (Figures SS9a and SS9b). If we scale $N_{e}$ by a factor of 10 to 25000, then the precision increased for $D^{*}$ to 37.56% and decreased for $D^{+}$ to 32.24% (Figures SS10e and SS10f). These results demonstrate that unequal genetic drift between populations do not have a large influence on $D^{*}$ and $D^{+}$.

### 3.3 Performance under aDNA Artifacts

**Figure 4:**
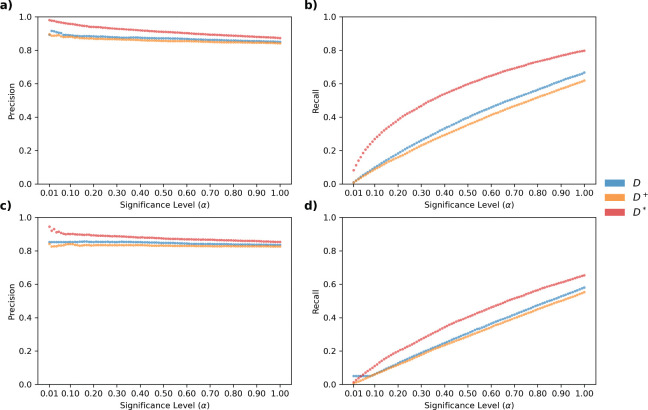
Precision and Recall in Presence of Simulated Missing Genotypes, Deamination, and Pseudohaploidzation Admixture proportion of f=0.03 was used. All statistics were computed in 50,000 Bp non-overlapping blocks. 12 diploids were sampled from $P_{1}$, 12 diploids were sampled from $P_{2}$, 2 diploids were sampled from $P_{3}$, and 1 diploid was sampled from $P_{4}$. **Panel a)** Precision and **Panel b)** recall are shown for significance levels (*α*) between 0.01 and 1. We introduced missing genotypes, deamination, and pseudohaploids (see Section 2.6) into the replicates and plotted the **Panel c)** Precision and **Panel d)** recall.

Simulations lack many of the errors found in aDNA. We simulated 10 diploid samples from $P_{1}$, 10 diploid samples from $P_{2}$, 2 diploid samples from $P_{3}$, and 2 diploid samples from $P_{4}$. We synthetically made all samples pseduohaploid and introduced synthetic deamination and missing genotypes. Typically, D and $D^{+}$ are used to test introgression between modern-day populations and an archaic population, where the archaic population’s genotypes are pseudohaploid and contain deamination and missing genotypes. We introduced these artifacts across all samples to maintain homogeneity. The precision and recall of the statistics are shown in Figures 4a and 4b. At α=0.05, we found that $D^{+}$ had a precision of 96.88% and a recall of 18.54%, while $D^{+}$ had a precision of 89.03% and a recall of 4.65%. Introducing pseudohaploids, deamination, and missing genotypes, $D^{*}$’s precision and recall dropped to 91.42% and 5.94%, respectively, and $D^{+}$’s precision and recall dropped to 83.24% and 3.02%, respectively.

We inspected the effects of pseudohaploids (Figure SS14), demaination (Figure SS15), and missing genotypes (Figure SS13) separately. We found that pseudohaploids had the greatest impact on precision compared to demaination and missing genotypes. At α=0.05, the introduction of pseudohaploids dropped the precision of $D^{*}$ to 90.22%. This is not surprising because pseudohaploids remove the additional information provided by heterozygous sites. Missing genotypes had the greatest impact on recall. At α=0.05, the introduction of missing genotypes decreased the recall of $D^{*}$ to 7.49%. Demaination had a negligible impact on the performance of $D^{*}$. Again, this is not surprising, since the large number of missing genotypes removes informative sites and increases the false-negative rate. The astute reader will notice that the performance of $D^{*}$ appears to be better when all three sources of error are present than when pseudohaploids are introduced alone. This is not the case, as the results shown in Figures 4c and SS14a were calculated from separate runs of EGGS [**?** ]. The results of pseudohaploids, deamination, and missing genotypes are meant to be compared to the simulated results without error (Figures 4a and 4b) and not in the presence of all three sources of error (Figures 4c and 4d).

### 3.4 Genome-wide Performance

We calculated the power of our simulations with a single lineage sampled from each population in the simplified demographic model (Figure 1) using a significance threshold of *p* < 0.05 (Figure SS16). The power of D was 73%, the power of $D^{+}$ was 52%, and the power of $D^{*}$ was 61%. A decrease in power shows an increase in the number of situations where there was genome-wide introgression, but the statistic was not deemed statistically significant. Patterson’s D comparatively has better power than $D^{+}$ and $D^{*}$ because the latter two incorporate ancestral allele sharing between populations. This could mask the genome-wide introgression signal.

For the cases of multiple lineages (N={2,5,10,25}), we calculated power similarly (Figure SS17). The power of D increased to 99%, the power of $D^{+}$ increased to 87%, and the power of $D^{*}$ increased to 99% when N=2 (Figure SS17a). This provides evidence that the rarefaction technique has some advantage over derived allele frequencies even for small sample sizes. All three statistics have power close to 100% when N=5 or greater (Figure SS17b, SS17c, SS17d). Our results show that $D^{*}$ can detect genome-wide introgression when multiple lineages are sampled from each population.

### 3.5 Denisovan Introgression in Papuans

$D^{*}$ is fundamentally an outlier test. Regions with a large $D^{*}$, in magnitude, compared to the genome-wide distribution could indicate that the region is introgressed. Therefore, $D^{*}$ and $D^{+}$ are exploratory statistics used to identify regions of interest for future analysis. We conducted our analysis on 27 Sardinian individuals, 17 Papuan individuals, and one Denisovan. The distribution of $D^{*}$ is shown in Figure SS19.

Table 1 shows the the genome-wide top 0.1% of blocks that contain genes. *RRM*1 [58], *HIC*2 [59], *BANK*1 [60], *CLEC*9*A* [61], and *LRBA* [62] are all related to immune function. This is consistent with expectations that genes related to the immune system are likely to be specifically introgressed from Denisovans into Papuans [53]. We found *ADK* (Adenosine Kinase), which is a potential candidate for positive selection in Papuans and thought to originate from archaic ancestors [63]. *ADK* is also related to immune function [64].

## 4 Discussion

Patterson’s D statistic is widely used to detect the presence of genome-wide archaic admixture [15]. Recent work has focused on identifying introgressed regions from archaic populations in modern-day populations [19, 17, 16]. In a three population tree with an unadmixed ancestral outgroup, Patterson’s D is calculated from the difference between the number of sites where the first and third population share the derived allele and the second and third population share the derived allele. An imbalance of counts indicates the presence of genome-wide introgression. D has a high false-positive rate in regions of low nucleotide diversity [19]. $D^{+}$ addressed this by incorporating sites where either pair of populations share the ancestral allele. When populations have multiple lineages, the counts are replaced with derived allele frequencies.

Here, we presented a new approach combining the simplicity of Patterson’s D [14] and the information captured by a rarefaction technique to count alleles private to pairs of populations [38]. Unlike D and $D^{+}$, our approach does not require an outgroup and can incorporate multiallelic sites. We evaluated D, $D^{+}$ [16], and $D^{*}$ using simulations generated under a simplified demographic model resembling archaic introgression (Figure 1). In most cases, $D^{*}$ performed better than or as well as $D^{+}$ despite varying mutation rate, recombination rate, and admixture proportion. $D^{*}$ showed an advantage over D and $D^{+}$ in the cases of single lineages and multiple lineages sampled from each population. Furthermore, $D^{*}$ comparatively maintained its performance when altering the level of drift each population experiences and the mutation rate within a specific population relative to another population. Finally, we examined $D^{*}$ in the presence of errors common to aDNA, such as pseudohaploids, deamination, and missing genotypes, and showed that while $D^{*}$ ’s performance did suffer, $D^{*}$ ’s inference is still reliable compared to other methods. In the case of determining the presence of genome-wide introgression, we caution against using $D^{*}$ when only single lineages are available from each population. If multiple lineages are sampled from each population, $D^{*}$ performs similarly to D and $D^{+}$ for detecting genome-wide introgression.

We evaluated $D^{*}$ on empirical samples by locating candidate regions of introgression between modern-day Papuans and Denisovans. We used $D^{*}$ as an exploratory method on additional lineages from Papuans and found genes such as *RRM*1, *HIC*2, *BANK*1, *CLEC*9*A*, and *LRBA*, which all have functions related to the immune system. This is consistent with previous analyses [23, 53]. In addition, we were able to recover regions belonging to *ADK*, a gene suspected for adaptation to high altitude [63].

For biallelic sites, an analogy holds between $D^{*}$ and $D^{+}$. It is worth discussing the reason for $D^{*}$ ’s performance gain over $D^{+}$. First, for the case of sampling single lineages from each population, $D^{*}$ showed a slight increase in precision compared to D and $D^{+}$. This occurs because $D^{*}$ is not restricted by an outgroup when counting sites with shared alleles between two populations. For example, if A is the ancestral allele and B is the derived allele, then $D^{+}$ will only count sites with *ABBA*, *BABA*, *BAAA*, and *ABAA*. $D^{*}$ will also count sites where where the first and third or the second and third population share the same allele, ignoring the allele’s state. These sites were avoided in the original definition of Patterson’s D because the additional sites artificially deflate the signal for genome-wide introgression between single lineages; however, in local regions, they provide useful information.

There are some points of consideration when using $D^{*}$. The first is common to many methods relying on a block (window) size. This is largely dependent on recombination rate and the estimated age of the potentially introgressed segments [30]. Without estimated divergence times between populations, this can be difficult. We suggest running $D^{*}$ multiple times with varying block sizes and looking for consistency in outlier regions. Finally, $D^{*}$ and $D^{+}$ provide an exploratory means of locating introgressed regions and lack measures of statistical significance in the absence of an underlying demographic model. Therefore, outlier regions identified with $D^{*}$ should be labeled as candidates for further analysis.

## Supplementary Material

Supplement 1

## Figures and Tables

**Table 1:** Top 0.1% of $D^{*}$ with Overlapping Genes for 27 Sardinian individuals, 17 Papuan individuals, and one Denisovan. Blocks without overlapping genes were removed.

Block	D*	Genes
chr1:100500127–100549923	0.28183	CDC14A,GPR88
chr2:15350020–15399910	0.402892	NBAS,RPS26P18
chr2:15400032–15449785	0.34397	NBAS
chr2:15450016–15499925	0.391287	NBAS
chr2:15500113–15549983	0.367268	NBAS
chr2:131300151–131349960	0.294214	PLEKHB2,FAR2P4,KLF2P4,ARHGAP42P1
chr2:151700003–151749947	0.277121	NEB
chr2:151750166–151800000	0.323337	ARL5A
chr2:224000182–224049908	0.257698	SERPINE2
chr3:51000035–51049848	0.377371	DOCK3
chr3:51050245–51099743	0.387618	DOCK3
chr4:16750069–16799774	0.29699	LDB2
chr4:101400095–101449946	0.258302	BANK1
chr4:107700145–107749836	0.290816	PAPSS1
chr4:123250242–123299824	0.258087	SPATA5
chr4:123300742–123349869	0.302236	SPATA5
chr4:150700338–150749602	0.275387	LRBA
chr4:151050038–151099860	0.251049	RPS3A
chr5:54800401–54849824	0.360699	CSPG4BP
chr6:159850073–159899838	0.285552	MAS1
chr7:96300201–96349542	0.354934	SLC25A13
chr8:145050202–145078629	0.250518	C8orf33
chr9:42850056–42899892	0.254684	ANKRD20A7P
chr9:42900101–42949610	0.296936	ANKRD20A7P,SNX18P5
chr10:19350216–19399928	0.257331	MALRD1
chr10:54250033–54299565	0.294402	PCDH15
chr10:73700011–73749562	0.291484	BMS1P4,GLUD1P3,DUSP8P5,SEC24C
chr10:74350281–74399904	0.256006	ADK,RPSAP6
chr10:74400047–74449866	0.273941	ADK,RAB5CP1
chr10:74450171–74499778	0.284567	ADK
chr11:4100485–4149958	0.247682	RRM1,OR55B1P
chr11:78500470–78549716	0.290268	NARS2
chr12:10050040–10099910	0.257952	CLEC9A,CLEC1A,
chr12:20850050–20899851	0.346022	SLCO1B3
chr12:87900259–87949986	0.257881	RPS4XP15
chr14:23450291–23499621	0.265123	NGDN
chr14:58250206–58299899	0.29471	ARMH4,PSMA3,HMGB1P14,ARID4A
chr14:60950594–60999149	0.253788	MNAT1,TRMT5,SLC38A6
chr15:45250073–45299715	0.245841	SLC28A2
chr20:37200293–37249953	0.295835	RPN2
chr22:21450168–21496820	0.266034	HIC2,TMEM191C,PI4KAP2
chr22:21500970–21547793	0.340411	PI4KAP2,RIMBP3C
