# Supplementary material for: A Rarefaction Approach to Identify Local Introgression in a Three Population Tree
Source: bioRxiv. 2026 May 15:2026.05.13.724952. Preprint. [Version 1] doi: 10.64898/2026.05.13.724952 (PMC13192780; doi:10.64898/2026.05.13.724952)
Supplement: Supplement 1 [file NIHPP2026.05.13.724952v1-supplement-1.pdf]

bioRxiv preprint doi: https://doi.org/10.64898/2026.05.13.724952; this version posted May 16, 2026. The copyright holder for this preprint (which was not certified by peer review) is the author/funder, who has granted bioRxiv a license to display the preprint in perpetuity. It is made available under a CC-BY 4.0 International license.

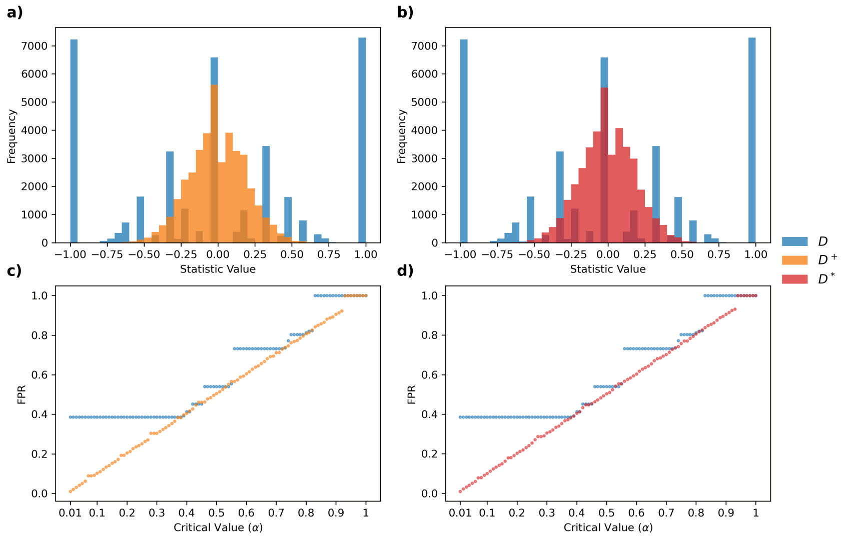

Figure S1: **Null Distribution and False-Positive Rates for Single Sampled Lineages.** Single lineage sampled from each population without introgression. All statistics were computed in 50,000 Bp non-overlapping blocks. **Panel a)** Null distribution for D^+ ($\mu = 0.0006, \sigma = 0.1894$). **Panel b)** Null distribution for D^* ($\mu = 0.0007, \sigma = 0.1838$). We display D's null-distribution for comparison and show it in blue ($\mu = 0.0018, \sigma = 0.6787$). **Panel c)** False-positive rate for D^+. **Panel d)** False-positive rate for D^*. False-positive rate for D is shown for comparison.

6 Supplemental

bioRxiv preprint doi: https://doi.org/10.64898/2026.05.13.724952; this version posted May 16, 2026. The copyright holder for this preprint (which was not certified by peer review) is the author/funder, who has granted bioRxiv a license to display the preprint in perpetuity. It is made available under a CC-BY 4.0 International license.

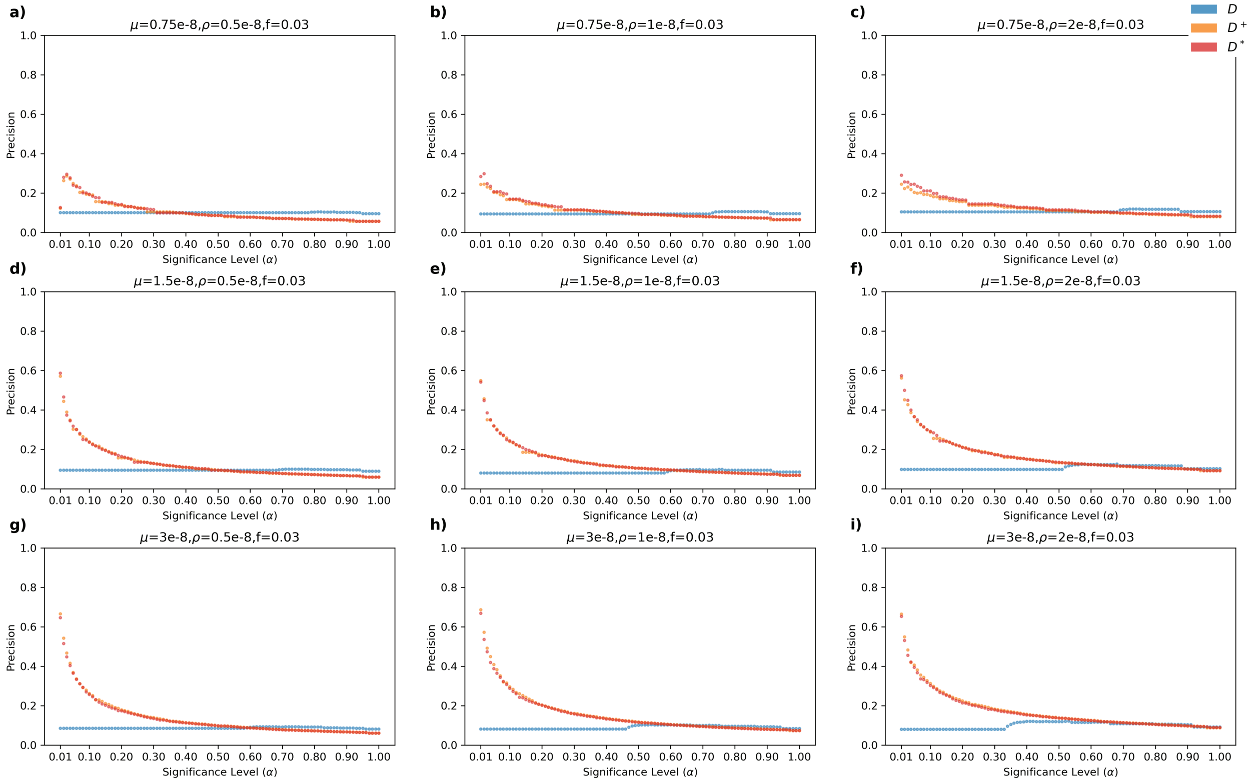

Figure S2: **Precision for $f = 0.03$ and Varying μ and ρ.** Admixture proportion of $f = 0.03$ was used. Single lineage sampled from each population. All statistics were computed in 50,000 Bp non-overlapping blocks. We show the precision for each pair of $\mu = \{0.75 \times 10^{-8}, 1.5 \times 10^{-8}, 3.0 \times 10^{-8}\}$ and $rho = \{0.5 \times 10^{-8}, 1 \times 10^{-8}, 1.5 \times 10^{-8}\}$ in **Panel a) - Panel i)**.

bioRxiv preprint doi: https://doi.org/10.64898/2026.05.13.724952; this version posted May 16, 2026. The copyright holder for this preprint (which was not certified by peer review) is the author/funder, who has granted bioRxiv a license to display the preprint in perpetuity. It is made available under a CC-BY 4.0 International license.

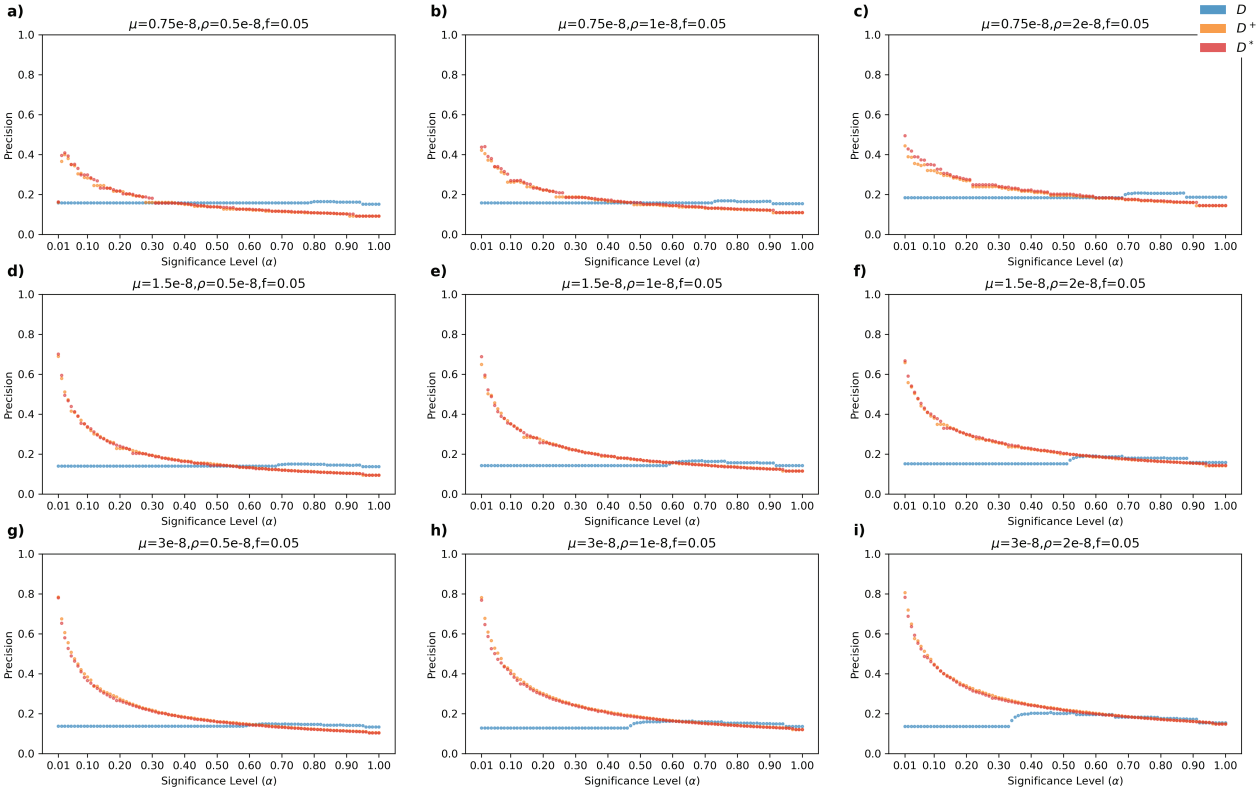

Figure S3: **Precision for $f = 0.05$ and Varying μ and ρ.** Admixture proportion of $f = 0.05$ was used. Single lineage sampled from each population. All statistics were computed in 50,000 Bp non-overlapping blocks. We show the precision for each pair of $\mu = \{0.75 \times 10^{-8}, 1.5 \times 10^{-8}, 3.0 \times 10^{-8}\}$ and $rho = \{0.5 \times 10^{-8}, 1 \times 10^{-8}, 1.5 \times 10^{-8}\}$ in **Panel a) - Panel i)**.

bioRxiv preprint doi: https://doi.org/10.64898/2026.05.13.724952; this version posted May 16, 2026. The copyright holder for this preprint (which was not certified by peer review) is the author/funder, who has granted bioRxiv a license to display the preprint in perpetuity. It is made available under a CC-BY 4.0 International license.

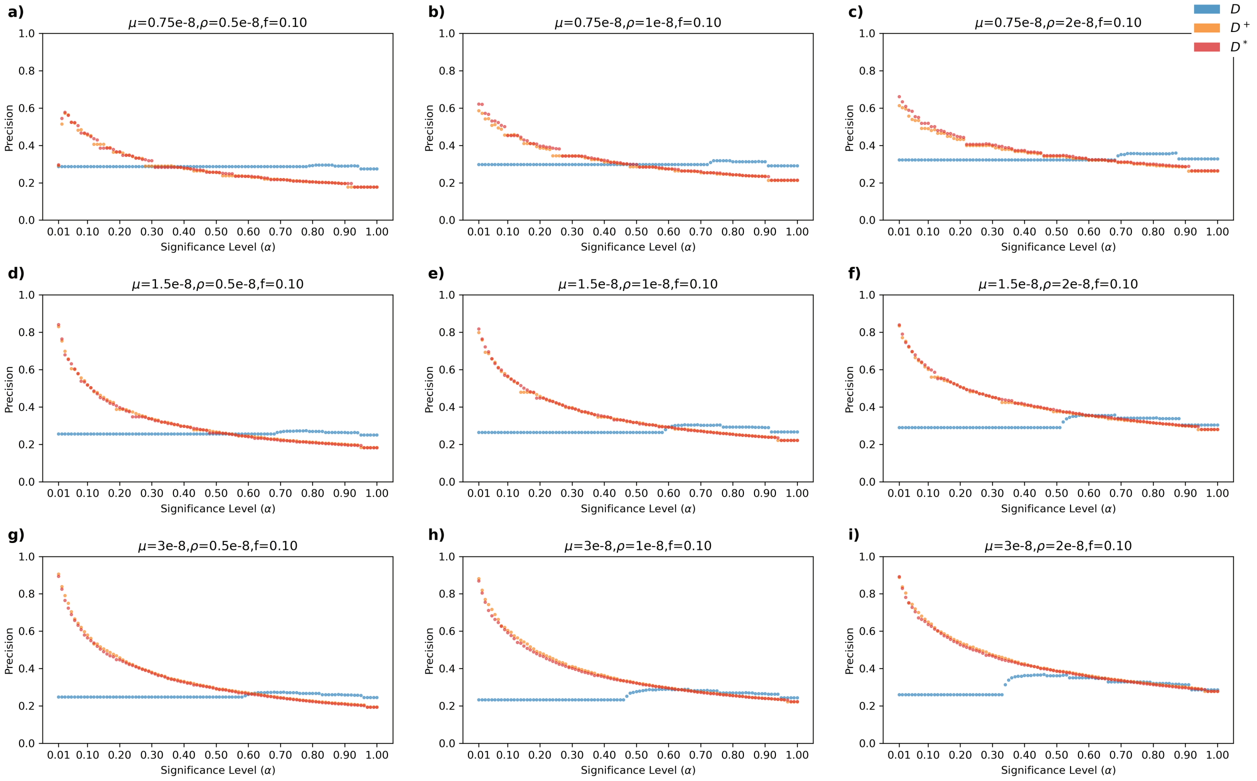

Figure S4: **Precision for $f = 0.10$ and Varying μ and ρ.** Admixture proportion of $f = 0.10$ was used. Single lineage sampled from each population. All statistics were computed in 50,000 Bp non-overlapping blocks. We show the precision for each pair of $\mu = \{0.75 \times 10^{-8}, 1.5 \times 10^{-8}, 3.0 \times 10^{-8}\}$ and $rho = \{0.5 \times 10^{-8}, 1 \times 10^{-8}, 1.5 \times 10^{-8}\}$ in **Panel a) - Panel i)**.

bioRxiv preprint doi: https://doi.org/10.64898/2026.05.13.724952; this version posted May 16, 2026. The copyright holder for this preprint (which was not certified by peer review) is the author/funder, who has granted bioRxiv a license to display the preprint in perpetuity. It is made available under a CC-BY 4.0 International license.

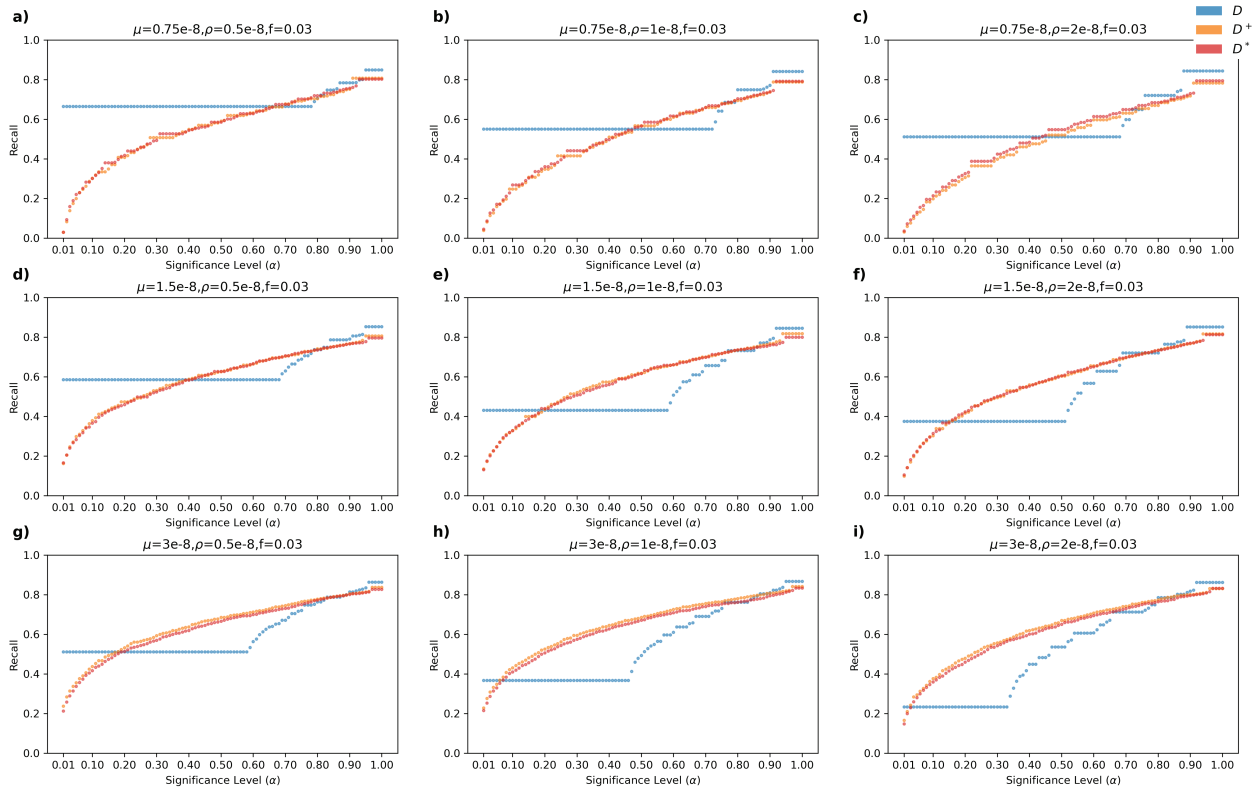

Figure S5: **Recall for $f = 0.03$ and Varying μ and ρ.** Admixture proportion of $f = 0.03$ was used. Single lineage sampled from each population. All statistics were computed in 50,000 Bp non-overlapping blocks. We show the recall for each pair of $\mu = \{0.75 \times 10^{-8}, 1.5 \times 10^{-8}, 3.0 \times 10^{-8}\}$ and $rho = \{0.5 \times 10^{-8}, 1 \times 10^{-8}, 1.5 \times 10^{-8}\}$ in **Panel a) - Panel i)**.

bioRxiv preprint doi: https://doi.org/10.64898/2026.05.13.724952; this version posted May 16, 2026. The copyright holder for this preprint (which was not certified by peer review) is the author/funder, who has granted bioRxiv a license to display the preprint in perpetuity. It is made available under a CC-BY 4.0 International license.

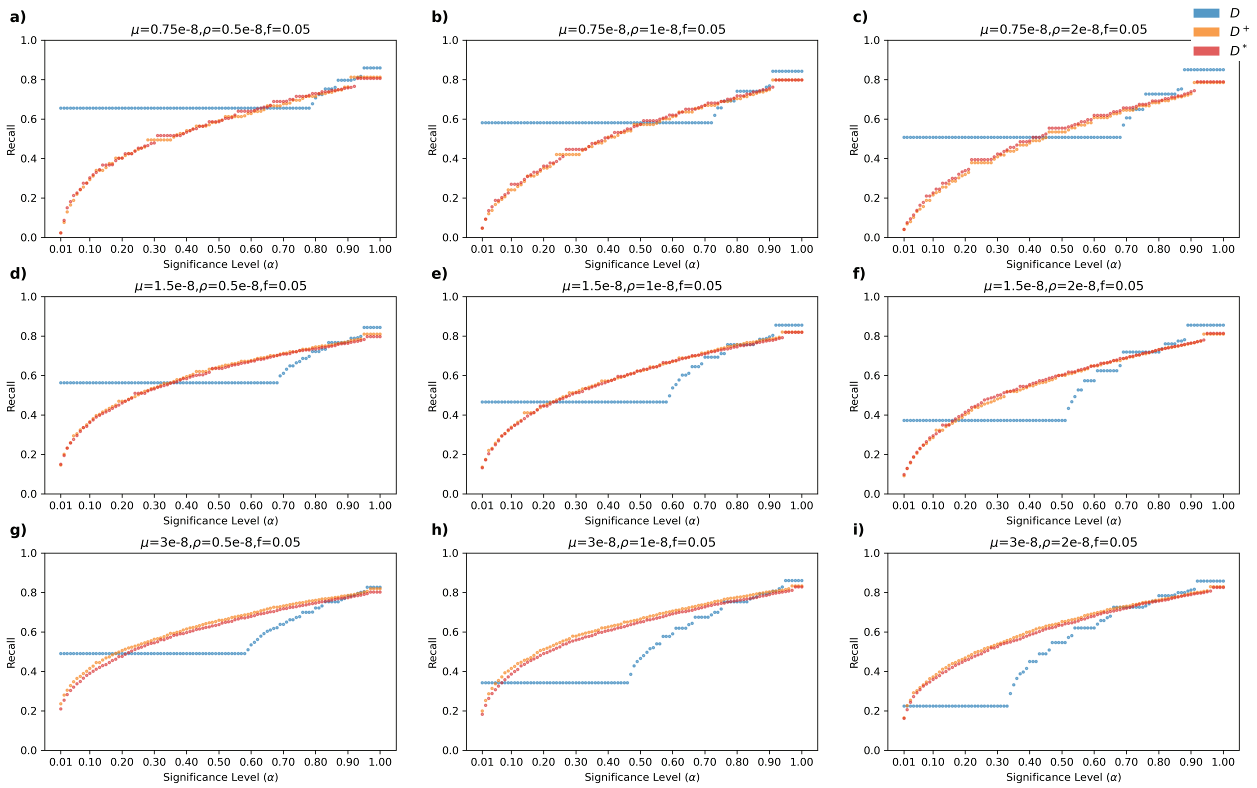

Figure S6: **Recall for $f = 0.05$ and Varying μ and ρ.** Admixture proportion of $f = 0.03$ was used. Single lineage sampled from each population. All statistics were computed in 50,000 Bp non-overlapping blocks. We show the recall for each pair of $\mu = \{0.75 \times 10^{-8}, 1.5 \times 10^{-8}, 3.0 \times 10^{-8}\}$ and $rho = \{0.5 \times 10^{-8}, 1 \times 10^{-8}, 1.5 \times 10^{-8}\}$ in **Panel a) - Panel i)**.

bioRxiv preprint doi: https://doi.org/10.64898/2026.05.13.724952; this version posted May 16, 2026. The copyright holder for this preprint (which was not certified by peer review) is the author/funder, who has granted bioRxiv a license to display the preprint in perpetuity. It is made available under a CC-BY 4.0 International license.

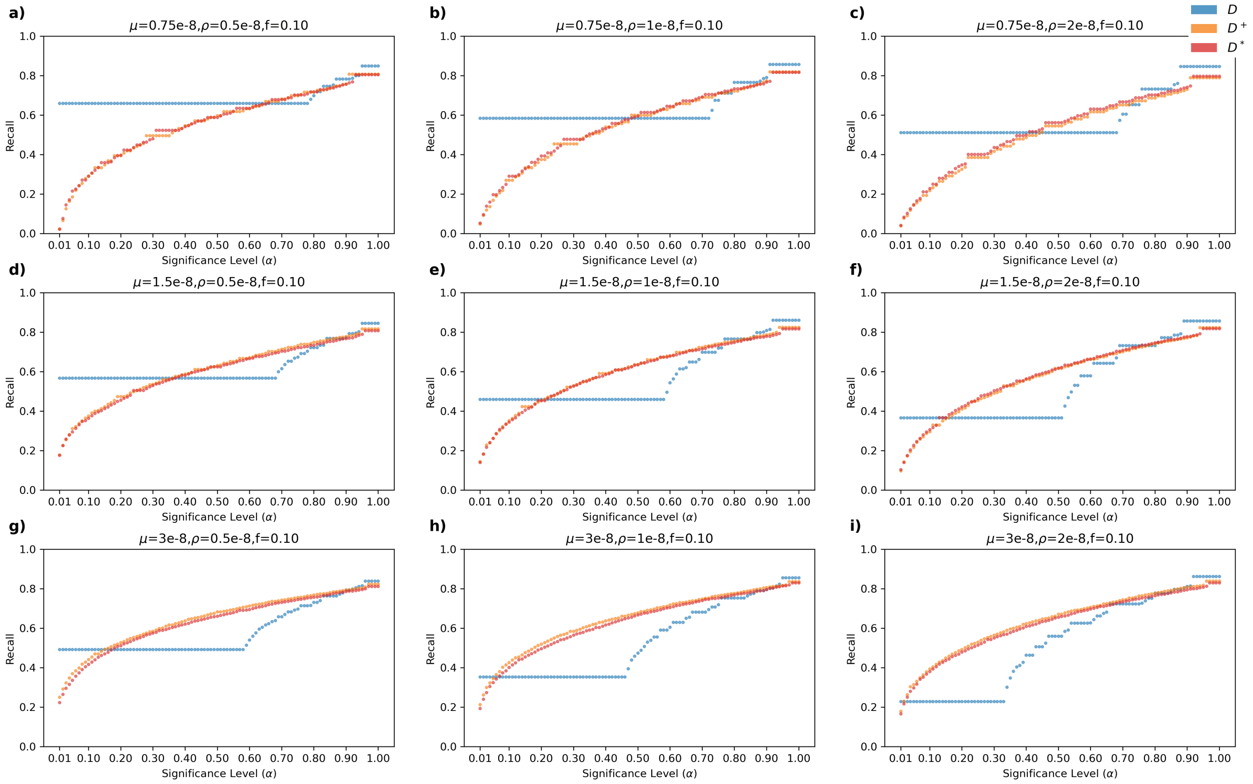

Figure S7: **Recall for $f = 0.10$ and Varying μ and ρ.**Admixture proportion of $f = 0.10$ was used. Single lineage sampled from each population. All statistics were computed in 50,000 Bp non-overlapping blocks. We show the recall for each pair of $\mu = \{0.75 \times 10^{-8}, 1.5 \times 10^{-8}, 3.0 \times 10^{-8}\}$ and $rho = \{0.5 \times 10^{-8}, 1 \times 10^{-8}, 1.5 \times 10^{-8}\}$ in **Panel a) - Panel i)**.

bioRxiv preprint doi: https://doi.org/10.64898/2026.05.13.724952; this version posted May 16, 2026. The copyright holder for this preprint (which was not certified by peer review) is the author/funder, who has granted bioRxiv a license to display the preprint in perpetuity. It is made available under a CC-BY 4.0 International license.

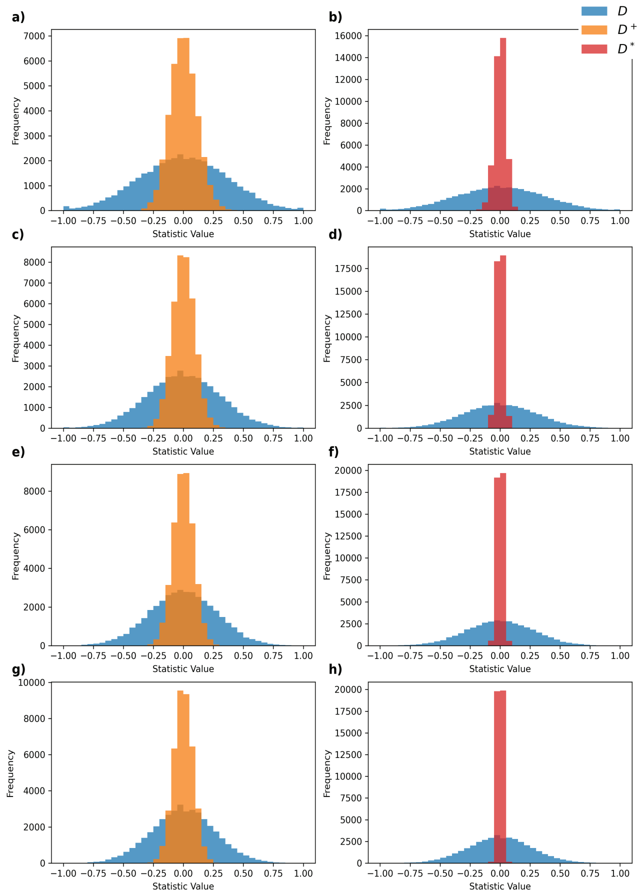

Figure S8: **Null Distribution for Multiple Sampled Lineages.** Multiple lineages sampled from each population without introgression. All statistics were computed in 50,000 Bp non-overlapping blocks. For each sample size, we show the null distribution for D in blue. **Panel a)** Null distribution for D^+ from 2 lineages sampled from P_2 ($\mu = 0.0027, \sigma = 0.1124$). **Panel b)** Null distribution for D^* from 2 lineages sampled from P_2 ($\mu = 0.0004, \sigma = 0.0452$). Null distribution for D ($\mu = -0.0010, \sigma = 0.3600$). **Panel c)** Null distribution for D^+ from 5 lineages sampled from P_2 ($\mu = 0.0016, \sigma = 0.0924$). **Panel d)** Null distribution for D^* from 5 lineages sampled from P_2 ($\mu = 0.0002, \sigma = 0.0276$). Null distribution for D ($\mu = 0.0006, \sigma = 0.3021$). **Panel e)** Null distribution for D^+ from 10 lineages sampled from P_2 ($\mu = 0.00009, \sigma = 0.0852$). **Panel f)** Null distribution for D^* from 10 lineages sampled from P_2 ($\mu = 0.0001, \sigma = 0.0224$). Null distribution for D ($\mu = 0.0005, \sigma = 0.2789$). **Panel g)** Null distribution for D^+ from 25 lineages sampled from P_2 ($\mu = 0.0002, \sigma = 0.0796$). **Panel h)** Null distribution for D^* from 25 lineages sampled from P_2 ($\mu = -0.00001, \sigma = 0.0179$). Null distribution for D ($\mu = -0.0012, \sigma = 0.2641$).

bioRxiv preprint doi: https://doi.org/10.64898/2026.05.13.724952; this version posted May 16, 2026. The copyright holder for this preprint (which was not certified by peer review) is the author/funder, who has granted bioRxiv a license to display the preprint in perpetuity. It is made available under a CC-BY 4.0 International license.

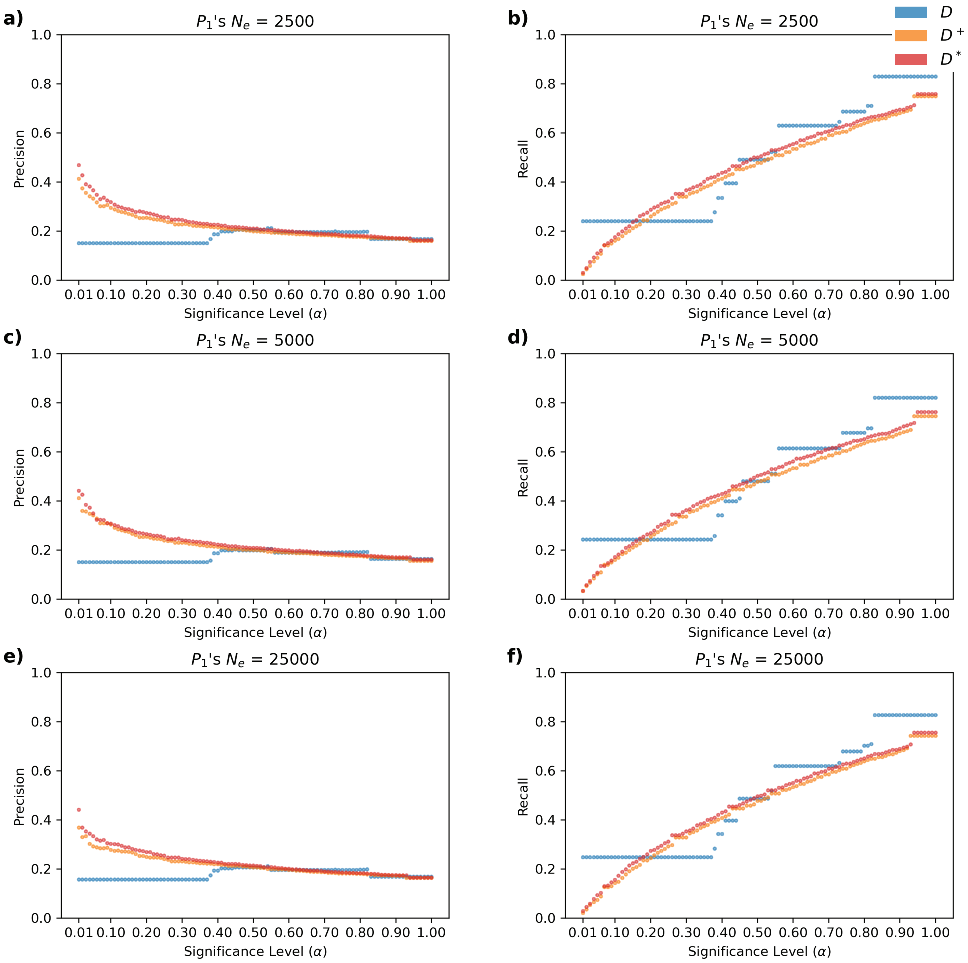

Figure S9: **Precision and Recall for Varying Drift in** P_1.Admixture proportion of $f = 0.10$ was used. Single lineage sampled from each population. All statistics were computed in 50,000 Bp non-overlapping blocks. We test the effects of drift by varying the effective population size for P_1. All other populations had an effective population size of 10000. **Panel a** Precision and **Panel b** recall for P_1's $N_e = 2500$, **Panel c** Precision and **Panel d** recall for P_1's $N_e = 5000$, and **Panel e** Precision and **Panel f** recall for P_1's $N_e = 25000$.

bioRxiv preprint doi: https://doi.org/10.64898/2026.05.13.724952; this version posted May 16, 2026. The copyright holder for this preprint (which was not certified by peer review) is the author/funder, who has granted bioRxiv a license to display the preprint in perpetuity. It is made available under a CC-BY 4.0 International license.

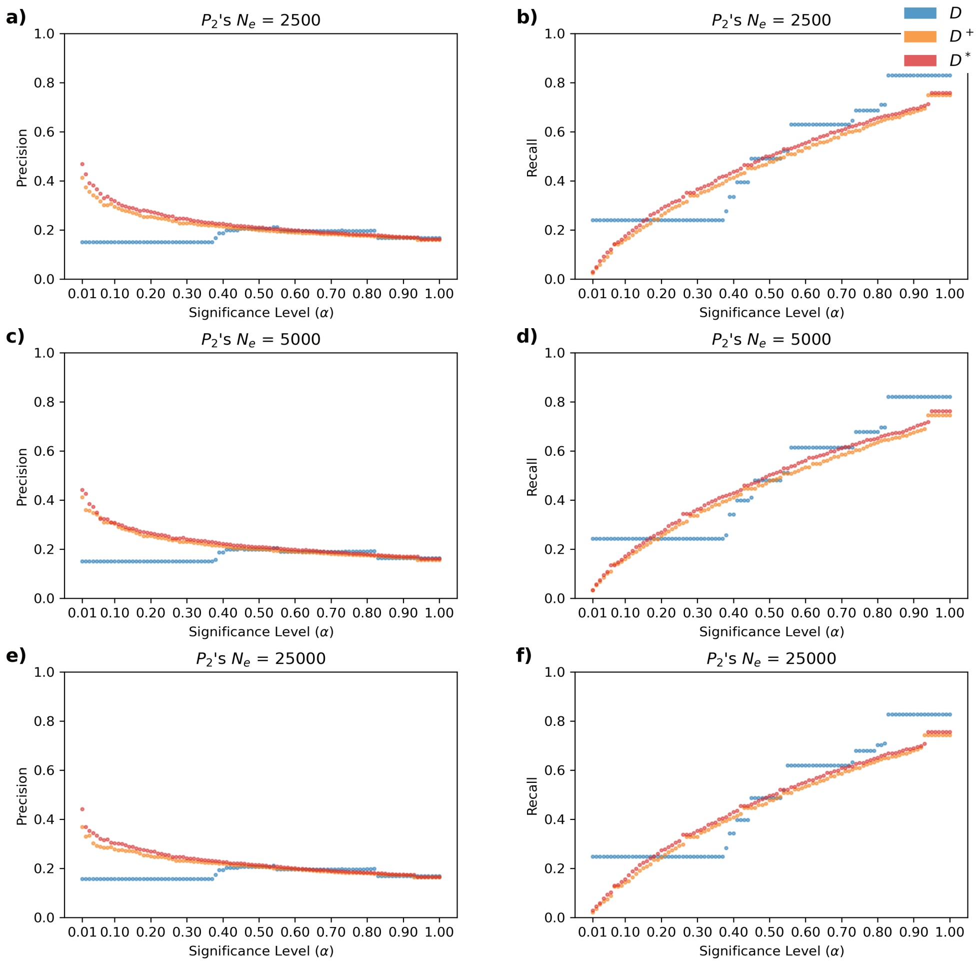

Figure S10: **Precision and Recall for Varying Drift in P_2.** Admixture proportion of $f = 0.10$ was used. Single lineage sampled from each population. All statistics were computed in 50,000 Bp non-overlapping blocks. We test the effects of drift by varying the effective population size for P_2. All other populations had an effective population size of 10000. **Panel a** Precision and **Panel b** recall for P_2's $N_e = 2500$, **Panel c** Precision and **Panel d** recall for P_2's $N_e = 5000$, and **Panel e** Precision and **Panel f** recall for P_2's $N_e = 25000$.

bioRxiv preprint doi: https://doi.org/10.64898/2026.05.13.724952; this version posted May 16, 2026. The copyright holder for this preprint (which was not certified by peer review) is the author/funder, who has granted bioRxiv a license to display the preprint in perpetuity. It is made available under a CC-BY 4.0 International license.

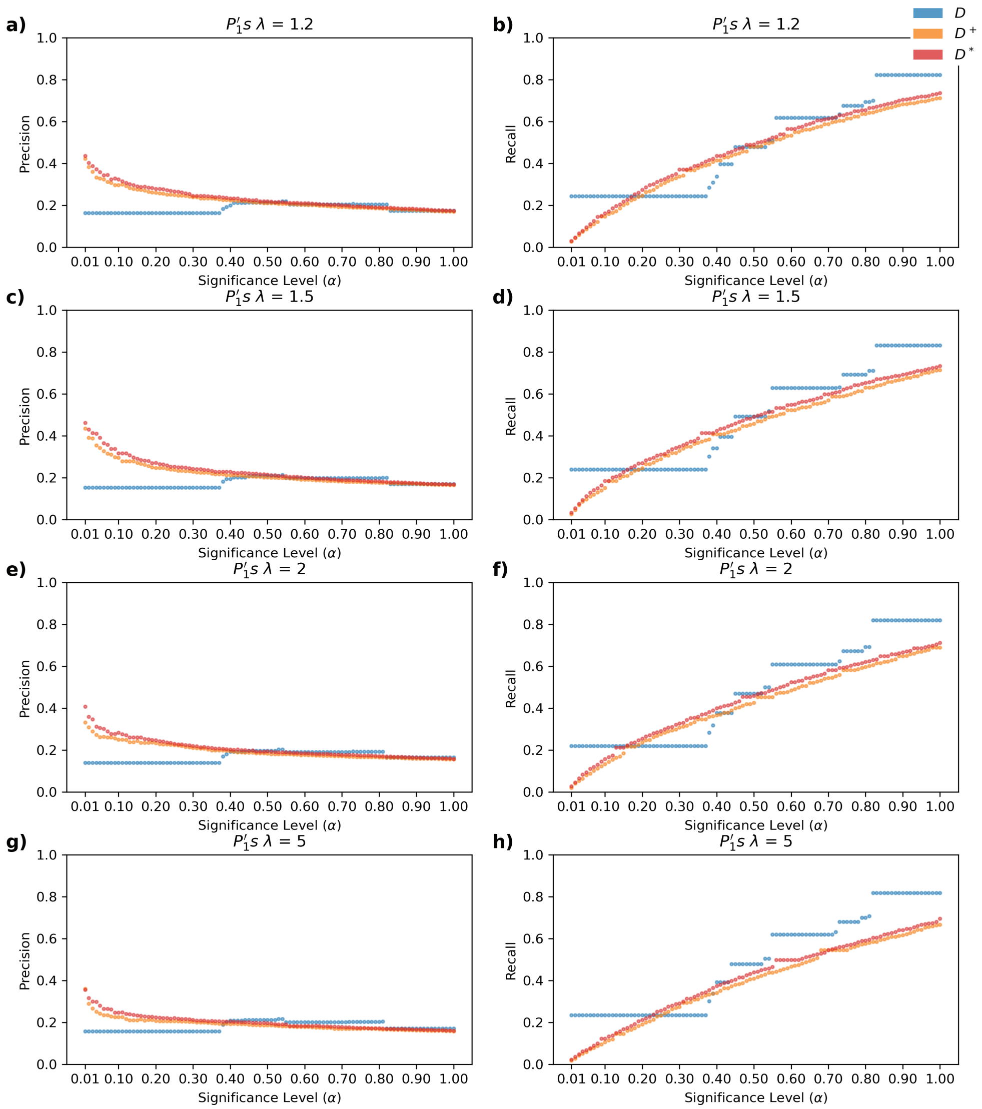

Figure S11: **Precision and Recall for Varying the Molecular Clock in P_1.** Admixture proportion of $f = 0.10$ was used. Single lineage sampled from each population. All statistics were computed in 50,000 Bp non-overlapping blocks. We test the effects of rescaling the mutation rate of P_1 by a factor of λ compared to the mutation rate of P_2. **Panel a** Precision and **Panel b** recall for $\lambda = 1.2$, **Panel c** Precision and **Panel d** recall for $\lambda = 1.5$, **Panel e** Precision and **Panel f** recall for $\lambda = 2$, and **Panel g** Precision and **Panel h** recall for $\lambda = 5$.

bioRxiv preprint doi: https://doi.org/10.64898/2026.05.13.724952; this version posted May 16, 2026. The copyright holder for this preprint (which was not certified by peer review) is the author/funder, who has granted bioRxiv a license to display the preprint in perpetuity. It is made available under a CC-BY 4.0 International license.

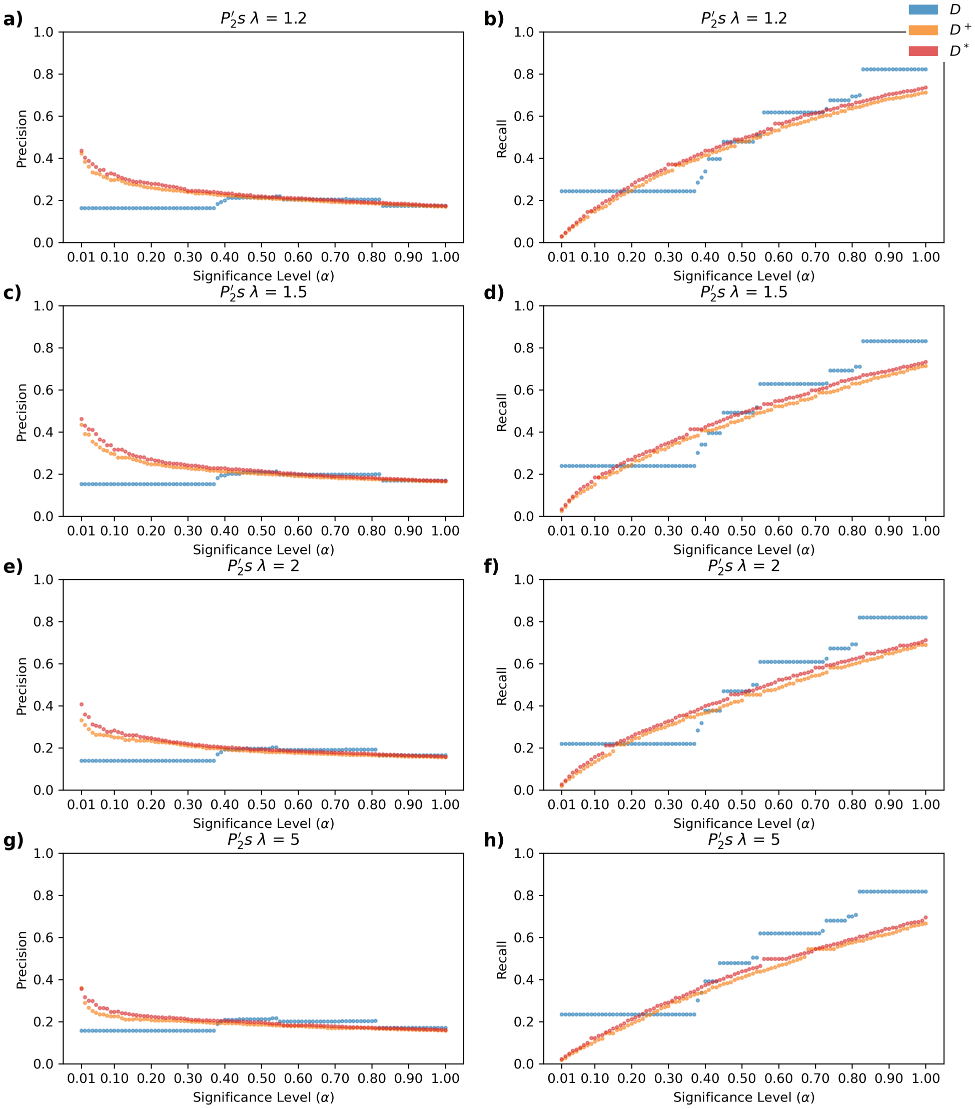

Figure S12: **Precision and Recall for Varying the Molecular Clock in P_2.** Admixture proportion of $f = 0.10$ was used. Single lineage sampled from each population. All statistics were computed in 50,000 Bp non-overlapping blocks. We test the effects of rescaling the mutation rate of P_2 by a factor of λ compared to the mutation rate of P_1. **Panel a** Precision and **Panel b** recall for $\lambda = 1.2$, **Panel c** Precision and **Panel d** recall for $\lambda = 1.5$, **Panel e** Precision and **Panel f** recall for $\lambda = 2$, and **Panel g** Precision and **Panel h** recall for $\lambda = 5$.

bioRxiv preprint doi: https://doi.org/10.64898/2026.05.13.724952; this version posted May 16, 2026. The copyright holder for this preprint (which was not certified by peer review) is the author/funder, who has granted bioRxiv a license to display the preprint in perpetuity. It is made available under a CC-BY 4.0 International license.

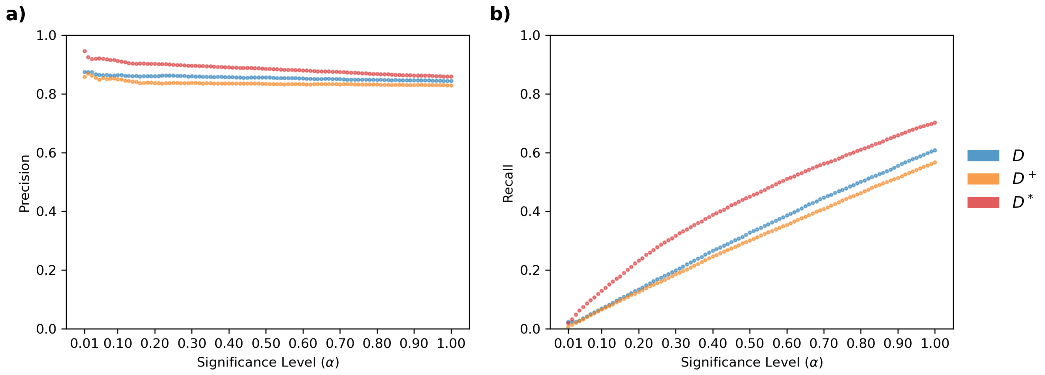

Figure S13: **Precision and Recall for Synthetic Missing Genotypes.** Admixture proportion of $f = 0.03$ was used. All statistics were computed in 50,000 Bp non-overlapping blocks. 12 diploids were sampled from P_1, 12 diploids were sampled from P_2, 2 diploids were sampled from P_3, and 1 diploid was sampled from P_4. Proportion of samples without either genotype at each site was drawn from a β-distribution with a mean of 0.55 and a standard deviation of 0.23. **Panel a** Precision and **Panel b** recall were plotted.

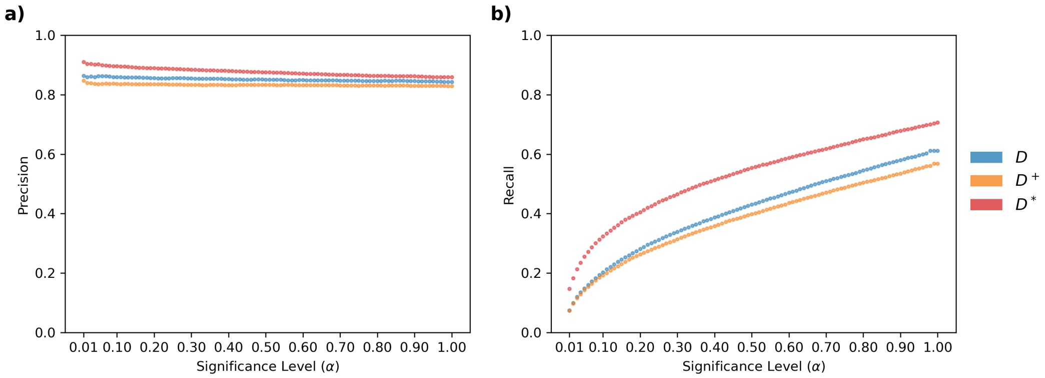

Figure S14: **Precision and Recall for Synthetic Pseudohaploidization.** Admixture proportion of $f = 0.03$ was used. All statistics were computed in 50,000 Bp non-overlapping blocks. 12 diploids were sampled from P_1, 12 diploids were sampled from P_2, 2 diploids were sampled from P_3, and 1 diploid was sampled from P_4. For every site, each heterozygous sample was chosen to be homozygous for the reference or alternative allele with equal probability. **Panel a** Precision and **Panel b** recall were plotted.

bioRxiv preprint doi: https://doi.org/10.64898/2026.05.13.724952; this version posted May 16, 2026. The copyright holder for this preprint (which was not certified by peer review) is the author/funder, who has granted bioRxiv a license to display the preprint in perpetuity. It is made available under a CC-BY 4.0 International license.

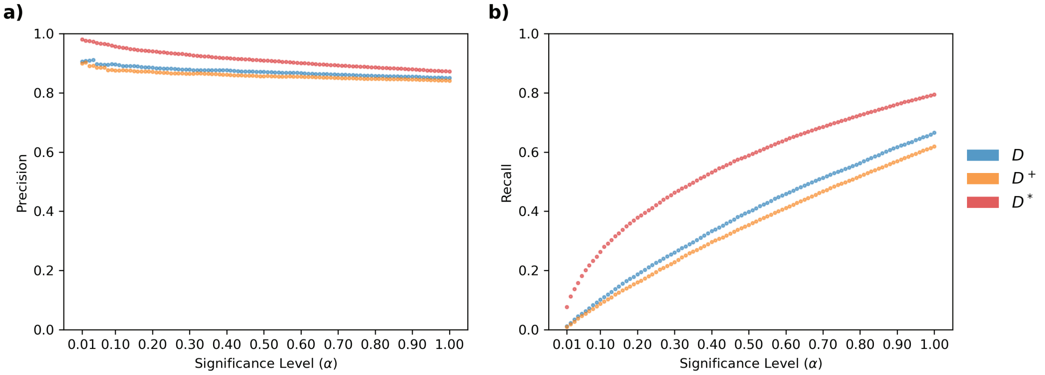

Figure S15: **Precision and Recall for Synthetic Deamination.** Admixture proportion of $f = 0.03$ was used. All statistics were computed in 50,000 Bp non-overlapping blocks. 12 diploids were sampled from P_1, 12 diploids were sampled from P_2, 2 diploids were sampled from P_3, and 1 diploid was sampled from P_4. Each site was a treated as a transition with a probability of 77.6%. Then, for every derived allele possessed by the samples, the allele was changed to the reference allele with a probability of 0.05%. **Panel a** Precision and **Panel b** recall were plotted.

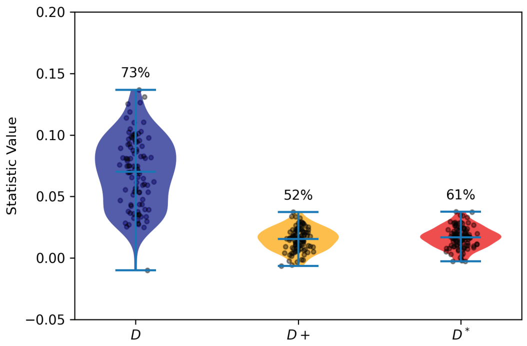

Figure S16: **Power for Single Sampled Lineages.** Admixture proportion of $f = 0.03$ was used. All statistics were computed in 50,000 Bp non-overlapping blocks. Block bootstrap calculated with 1000 iterations. Power was calculated using a significance threshold of $p > 0.05$ and is shown above each violin plot. Black dots indicate the value of the statistic, and the bars show the minimum, the maximum, and the mean value.

bioRxiv preprint doi: https://doi.org/10.64898/2026.05.13.724952; this version posted May 16, 2026. The copyright holder for this preprint (which was not certified by peer review) is the author/funder, who has granted bioRxiv a license to display the preprint in perpetuity. It is made available under a CC-BY 4.0 International license.

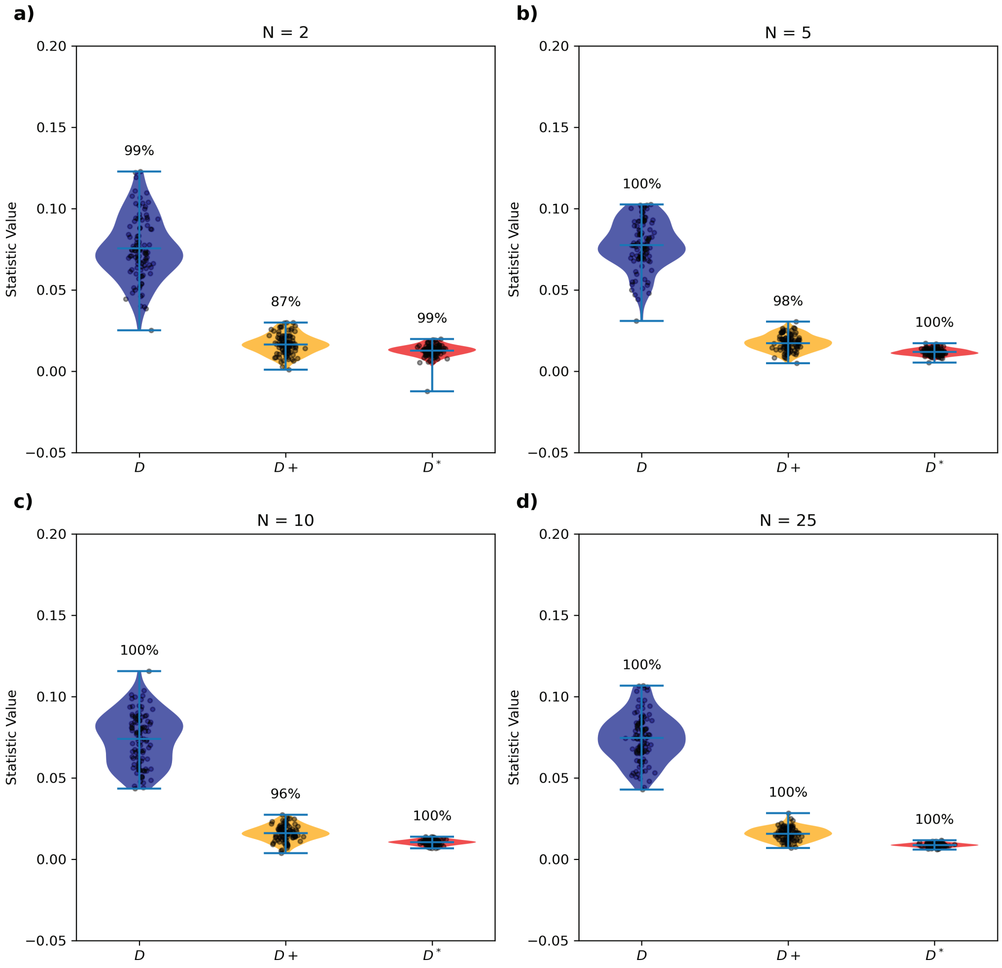

Figure S17: **Power for Multiple Sampled Lineages.** Admixture proportion of $f = 0.03$ was used. All statistics were computed in 50,000 Bp non-overlapping blocks. 25 lineages were sampled from P_1, 2 lineages were samples from P_3, and 2 lineages were sampled from P_4. The number of lineages sampled from P_2 were varied for $N = \{2, 5, 10, 25\}$. Block bootstrap calculated with 1000 iterations. Power was calculated using a significance threshold of $p > 0.05$ and is shown above each violin plot. Black dots indicate the value of the statistic, and the bars show the minimum, the maximum, and the mean value. **Panel a)** Power for $N = 2$. **Panel b)** Power for $N = 5$. **Panel c)** Power for $N = 10$. **Panel d)** Power for $N = 25$.

bioRxiv preprint doi: https://doi.org/10.64898/2026.05.13.724952; this version posted May 16, 2026. The copyright holder for this preprint (which was not certified by peer review) is the author/funder, who has granted bioRxiv a license to display the preprint in perpetuity. It is made available under a CC-BY 4.0 International license.

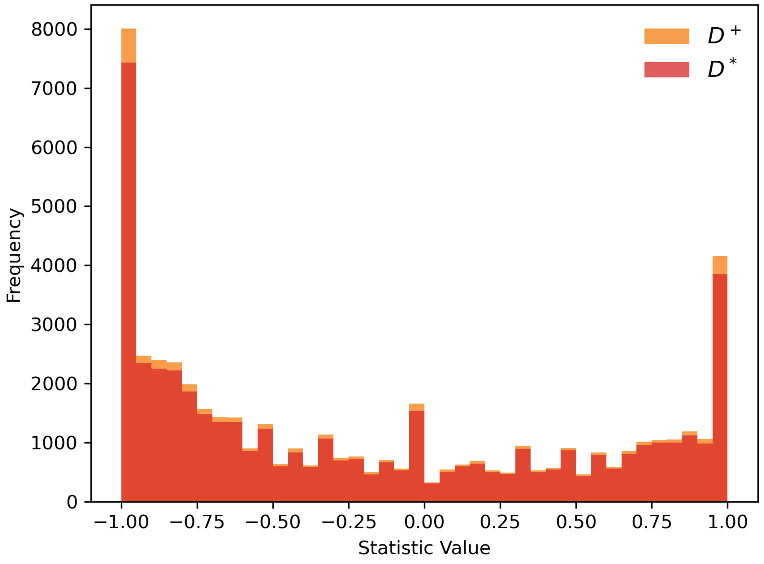

Figure S18: **Distribution of D^* and D^+ for Denisovan Introgression with $N = 1$.** A single lineage was sampled from Sardinians, Papuans, and Denisovans. D^* and D^+ were calculated in 50 Kb blocks. D^* mean is -0.1957 and standard deviation is 0.7098. D^+ mean is -0.1960 and standard deviation is 0.7107.

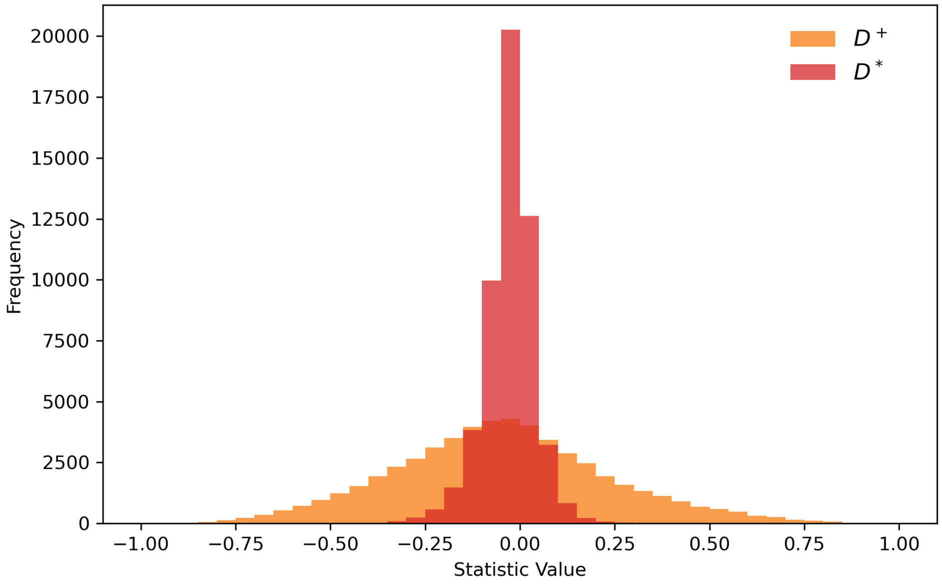

Figure S19: **Distribution of D^* and D^+ for Denisovan Introgression with Multiple Sampled Lineages.** All samples are diploid. 27 Sardinian individuals, 17 Papuan individuals, and one Denisovan were sampled. D^* and D^+ were calculated in 50 Kb blocks. D^* mean is -0.0277 and standard deviation is 0.0669. D^+ mean is -0.0499 and standard deviation is 0.2843.
